# Transcriptome Profiles of IncRNA and mRNA Highlight the Role of Ferroptosis in Chronic Neuropathic Pain With Memory Impairment

**DOI:** 10.3389/fcell.2022.843297

**Published:** 2022-04-25

**Authors:** Yidan Tang, Changliang Liu, Tao Zhu, Hai Chen, Yalan Sun, Xueying Zhang, Qi Zhao, Jiahui Wu, Xuejie Fei, Shixin Ye, Chan Chen

**Affiliations:** ^1^ Department of Anesthesiology and National Clinical Research Center for Geriatrics, Laboratory of Anesthesia and Critical Care Medicine, Translational Neuroscience Center, West China Hospital, The Research Units of West China, Chinese Academy of Medical Science, Sichuan University, Chengdu, China; ^2^ Precision Medicine Research Center, West China Hospital, Sichuan University, Chengdu, China; ^3^ Department of Anesthesiology and Perioperative Medicine, Shanghai Fourth People’s Hospital Affiliated to Tongji University School of Medicine, Shanghai, China; ^4^ Unité INSERM U1195, Hôpital de Bicêtre, Le Kremlin-Bicêtre, Université Paris-Saclay, Paris, France

**Keywords:** pain, memory impairment, RNA-seq, ferroptosis, ATF3

## Abstract

**Background:** Chronic neuropathic pain is commonly associated with memory loss, which increases the risk of dementia, lowers life quality and spending. On the other hand, the molecular processes are unknown, and effective therapies have yet to be discovered. Long non-coding RNAs (lncRNAs) are emerging potential therapeutic targets for chronic pain, but their role in chronic pain-induced memory impairment is unknown.

**Methods:** We established a CCI-induced memory impairment rat model. To investigate and validate the gene expression alterations in the hippocampus of CCI-induced memory impairment, we used RNA-Seq, bioinformatics analysis, qRT-PCR, western blot, immunostaining, Nissl staining, and Diaminobenzidine-enhanced Perls’ stain.

**Results:** CCI rats displayed long-term memory deficits in the Y maze and novel objective recognition tests, and chronic mechanical and thermal pain hypersensitivity in the hind paws. We found a total of 179 differentially expressed mRNAs (DEmRNAs) (81 downregulated and 98 upregulated) and 191 differentially expressed long noncoding RNAs (DElncRNAs) (87 downregulated and 105 upregulated) between the hippocampus CA1 of CCI-induced memory impairment model and the sham control, using RNA-Seq expression profiles. The most enriched pathways involving oxidation and iron metabolism were explored using a route and function pathway analysis of DEmRNAs and DElncRNAs. We also discovered that ATF3 was considerably overexpressed in the hippocampal CA1 area, and gene markers of ferroptosis, such as GPX4, SLC7A11, SLC1A5, and PTGS2, were dysregulated in the CCI-induced memory impairment paradigm. Furthermore, in the hippocampus CA1 of CCI-induced memory impairment, lipid peroxidation and iron overload were considerably enhanced. Fer-1 treatment reversed ferroptosis damage of CCI with memory impairment model. Finally, in CCI-induced memory impairment, a competing RNA network analysis of DElncRNAs and DEmRNAs was performed to investigate the putative regulatory link of DElncRNAs on DEmRNAs via miRNA sponging.

**Conclusion:** Using RNA-Seq, we created a genome-wide profile of the whole hippocampus of a rat model of CCI-induced memory impairment. In the hippocampus, pathways and function analyses revealed numerous intriguing genes and pathways involved in ferroptosis and memory impairment in response to chronic pain stress. As a result, our research may aid in the identification of potential and effective treatments for CCI-induced memory impairment.

## 1 Introduction

Memory impairment is one of the most common problems in the context of chronic pain stress, mainly manifesting as impairments in attention, learning memory, information processing speed, and executive capacity, and its long-term progression raises the risk of dementia (Dick and Rashiq, 2007; Moriarty et al., 2011; Whitlock et al., 2017; Mazza et al., 2018; Zhang et al., 2021). Working memory problems are present in 70% of people with chronic pain worldwide (Berryman et al., 2013). In recent clinical investigations, patients with peripheral neuropathic pain have objective cognitive deficits (Ojeda et al., 2018; Tyrtyshnaia and Manzhulo, 2020). Furthermore, chronic pain with memory loss has been linked to a lower quality of life and increased spending (Whitlock et al., 2017). However, the cause of pain-related cognitive impairment is unknown, and there is no effective prevention or treatment for chronic pain-related cognitive impairment. Analgesic medications routinely used in clinics can help with pain relief, but they can also cause and/or exacerbate the cognitive dysfunction that comes with it (Moriarty et al., 2011). Therefore, exploring the etiology and preventative strategies of chronic pain accompanied by cognitive impairment is crucial in therapeutic practice.

Apoptosis, autophagy, and ferroptosis are cell death processes with diverse morphologies and molecular properties that play an essential role in the pathology of neurodegenerative disorders (Moujalled et al., 2021). The accumulation of lipid peroxidation products and fatal reactive oxygen species characterizes ferroptosis, an iron-dependent, programmed form of cell death (Dixon et al., 2012). A growing body of evidence suggests that aberrant iron metabolism plays a crucial role in Parkinson’s disease, Alzheimer’s disease, Huntington’s disease, amyotrophic lateral sclerosis, and cerebral hemorrhage models (Stockwell et al., 2017). A growing body of evidence suggests that aberrant iron metabolism plays a crucial role in Parkinson’s disease, Alzheimer’s disease, Huntington’s disease, amyotrophic lateral sclerosis, and cerebral hemorrhage models (Stockwell et al., 2017). Moreover, researchers discovered a decreased GPX4 level and an increased ACSL4 expression level (markers of ferroptosis) in the spinal cords of chronic pain model rats and mice and aberrant iron deposition (Wang H et al., 2021). Furthermore, the ferroptosis inhibitors were revealed to effectively reduce pain behavior, implying that ferroptosis may play a role in chronic pain. Moreover, the ferroptosis inhibitors were found to effectively reduce pain behavior, indicating that ferroptosis may play a role in chronic pain (Guo et al., 2021). However, it is unclear whether ferroptosis is associated with memory impairment in patients suffering from chronic pain.

Long non-coding RNAs (lncRNAs) are a type of non-coding transcript that is longer than 200 nucleotides (Bhat et al., 2016), exhibited a potential and multifunctional regulation in gene expression (Ulitsky and Bartel, 2013). Following peripheral nerve injury, lncRNA expression was found to be dysregulated in nerves (Chen Y et al., 2021), the dorsal root ganglion (DRG) (Pan et al., 2021), and the spinal cord (Wu et al., 2021). While researchers are increasingly focused on the role of lncRNA in chronic pain, the significance of lncRNA in the comorbidity of chronic pain and cognitive impairment remains unknown.

To learn more about the biological pathways that cause chronic pain-induced memory impairment. In a chronic constriction injury (CCI)-induced memory impairment rat paradigm, we performed a genome-wide RNA-Seq (mRNAs and lncRNAs). We also looked into the critical pathological pathways in which differentially expressed genes (DEGs) have a role. Our findings could lead to more research into the intriguing genes or signaling pathways that protect against memory loss caused by prolonged stress.

## 2 Materials and Methods

### 2.1 Animals Preparation and Groups

Male Sprague–Dawley rats (5–8 weeks, 180–220 g) were purchased from Chengdu Dossy Experimental Animals CO., LTD. (Chengdu, Sichuan, China) and maintained under controlled conditions with no restrictions on food or water on a 12 h light/dark cycle. The rats were given some time to adjust to their new surroundings before the experiment. The Animal Care and Use Committee at Sichuan University approved all of the experimental techniques. The experiments *in vivo* were performed in 3 parts. In the first part, rats were randomly divided into Sham and CCI groups for the behavior tests, RNA-seq, and molecular and biochemical analysis. In the second part, rats received CCI induction for validation of transcriptome profiles, detection of iron content, and lipid peroxidation levels. In the third part, rats were divided into the Sham + vehicle group, CCI group, CCI + vehicle group, CCI + Ferrostatin-1 (Fer-1) group. Fer-1 was dissolved in 1% dimethyl sulfoxide (DMSO). For CCI + Fer-1, rats were administered with Fer-1 2 h after model establishment (intraperitoneal injections (i.p.), 2 mg/kg), followed three times a week for 21 days. For the Sham + vehicle group and CCI + vehicle group, rats receive an equal volume of 1% DMSO solvent.

### 2.2 Model Establishment

The CCI model was established based on the findings of a prior study (Bennett and Xie, 1988). Briefly, the left sciatic nerve was exposed at mid-thigh level after rats were sedated and maintained with 2–3% isoflurane. The nerve trunk was then loosely tied four times using 4-O gut sutures near the branching site. Only anesthesia, incision, and nerve exposure were given to the sham rats. The rats eventually recovered on a heating pad and were returned to their cages.

### 2.3 Behavior Tests

#### 2.3.1 Mechanical allodynia

As previously reported, von Frey filaments were used to assess mechanical allodynia. Before the experiment, the rats were placed for 30 min in a transparent glass frame (22 × 10 × 14 cm). The up-down method used six precisely calibrated von Frey filaments to test the paw withdrawal thresholds (PWTs). Von Frey fiber filaments stimulated the plantar of the rat hind paw in a perpendicular manner (range from 0.008 to 300 g). Retraction of the foot, withdrawal or paw licking were all considered positive responses.

#### 2.3.2 Thermal Hyperalgesia

Thermodynamic hyperalgesia was measured using the approach previously described. In addition, the paw withdrawal latencies (PWLs) were measured using a thermal pain stimulator (Ugo Basile business). Three trials with a 5-min gap were given to each rat. The 20 s were selected as a stimulation cut-off time to avoid tissue damage.

#### 2.3.3 Cold Test

As described previously, the cold test was carried out (You et al., 2018), with acetone as the solvent. A hind paw’s lateral plantar surface was treated with 50 μl of acetone. Bisk foot removal was considered a positive response. Both the paw withdrawal score and the length of the withdrawal were recorded.

#### 2.3.4 Y Maze Test

The Y maze test was used to examine spontaneous modification behavior. The equipment consisted of three identical arms with a 120° angle, designated arm A, B, and C (50 cm in length, 10 cm in width, 25 cm in height). Each animal was carefully placed in the maze’s center, then tracked and filmed for 8 min using a video-tracking device. A spontaneous alternation was defined as entering all three arms in the consecutive order as ABC, BCA, and CAB. The following calculation was used to calculate the percentage of spontaneous alternation: percent spontaneous alternation = number of spontaneous alternations/(total arm entries—2) *100.

#### 2.3.5 Novel Objective Recognition Test

The NOR test was carried out in a box measuring 60 × 60 × 50 cm. The experiment was split into two parts: training and testing. Each animal was placed in a quiet experimental area for at least 30 min before the test. For the training step, each rat was placed in a box with two identical objects for 10 minutes. Each rat was placed in the box for 5 min after a 24-h intertrial interval in the home cage, where one of the familiar objects was swapped with a novel object. Exploratory activity was characterized as sniffing or touching the object, and the total time spent investigating within 1.5 cm for each object was recorded. As a result, the new item recognition index is calculated as follows: new object recognition index = (time spent investigating the novel object/total time spent exploring the novel and familiar objects).

### 2.4 Tissue Collection and RNA Extraction

The rats were sedated with sodium pentobarbital (40 mg/kg, i. p.) on day 21, and the rat’s entire hippocampus was promptly collected under the ice. According to the manufacturer’s protocol, the mirVanaTM miRNA ISOlation Kit (Ambion) was used to extract total RNA from the hippocampus of the CCI and sham groups. The Agilent 2,100 Bioanalyzer assessed RNA integrity (Agilent Technologies, Santa Clara, CA, United States). The samples with an RNA Integrity Number (RIN) of 7 were sent for further testing.

### 2.5 RNA-Seq Library Establishment and RNA-Seq

The libraries were built using TruSeq Stranded Total RNA with Ribo-Zero Gold, as directed by the manufacturer. To Ribo-Zero deplete and fragment RNA, about 1 g total RNA was processed using TruSeq Stranded Total RNA with Ribo-Zero Gold Kit. After that, the first-strand cDNA synthesis was treated with Act D Mix, followed by the second-strand cDNA synthesis. The 3′ends of cDNA were then adenylated, and the adapters were ligated to the 3′adenylated ends. The PCR Primer Cocktail and PCR Master Mix were also employed to enrich cDNA fragments. The size and purity of the sample were checked using an Agilent Technologies 2,100 Bioanalyzer. Finally, these libraries were sequenced on an Illumina sequencing device (HiSeqTM 2,500 platform), and paired-end reads of 150 bp/125 bp were generated.

### 2.6 Bioinformatics Analysis and Differentially Expressed Genes Analysis

Quality control (QC) was performed on the primary sequencing data generated by RNA-Seq (raw reads). Table 1 summarizes the details of total readings and mapping ratio reads. By deleting the adaptor, low-quality bases, low-quality reads, and reads with unknown N bases, Trimmomatic software (Bolger et al., 2014) filtered the raw reads into high-quality clean reads. HISAT2 (version 2.2.1.0) (Kim et al., 2015) was used to align the clean reads to the reference genome and the RSeQC (version 2.6.4) (Wang et al., 2012) technique was used to count the proportions of various comparison types and evaluate the comparison results. Stringtiew2 software (version 1.3.3b) (Pertea et al., 2015) was used to assemble the reads and splice the new transcript after getting the SAM file with the comparison findings. The candidate lncRNA transcripts were then chosen by comparing the gene annotation information of the reference sequence obtained by Cuffcompare software (version 2.2.1) to the gene annotation information of the candidate lncRNA transcripts (Trapnell et al., 2012). Finally, to acquire lncRNA predicted sequences, transcripts with coding potential were screened out using CPC (Kong et al., 2007), Pfam (version v30) (Finn et al., 2006), and PLEK (version 1.2) (Li et al., 2014). The sequencing reads were aligned with the sequence of mRNA transcript sequences using Bowtie (version 2.2.9) (Langmead and Salzberg, 2012). And then, eXpress (version 1.5.1) (Roberts and Pachter, 2013) was applied to make quantitative gene analysis, the FPKM value, and counts value. Finally, differential expression analysis was done using DESeq2 (version 1.18.0) (Delhomme et al., 2012) with a *p*-value of 0.05 and |Log2 (fold change)| of 0.58.

**TABLE 1:** The total reads and mapping ratio for sham and CCI with memory impairment groups by RNA-Seq.

Sample	Total raw reads (Mb)	Total clean reads (Mb)	Clean reads Q30 (30%)	Valid bases (%)	Total mapped reads (%)
Sham 1	94.89	93.35	93.33	92.78	96.61
Sham 2	99.48	97.87	93.32	93.03	96.52
Sham 3	97.62	96	93.31	92.22	96.46
CCI 1	95.85	94.25	93.01	92.92	96.42
CCI 2	94.85	93.36	93.13	92.84	96.4
CCI 3	96.6	94.98	93.38	92.66	96.33

### 2.7 Functional Enrichment Analysis of Differentially Expressed Genes

The detected DEGs were subjected to gene ontology (GO) (http://geneontology.org/) and Kyoto Encyclopedia of Gene and Genomes (KEGG) (http://www.genome.jp/kegg/) enrichment analysis based on the hypergeometric distribution test. Biological process (BP), cellular component (CC), and molecular function were all included in the GO study (MF). The enrichment scores were used to rank the KEGG analysis pathways.

### 2.8 Real-Time qPCR

Rather than using total RNA from the hippocampus for RNA-Seq, HiScript^®^ III-RT SuperMix (Vazyme Biotech Co., Ltd., China) was used to reverse transcribe total RNA from the hippocampus into cDNA according to the manufacturer’s instructions. Table 2 lists all of the primer sequences used. As an internal reference gene, 18sRNA was employed. The qRT-PCR was performed using a Taq Pro Universal SYBR qPCR Master Mix (Vazyme Biotech Co., Ltd., China) kit with a 20L reaction system and a CFX96 Real-Time System (Bio-Rad Laboratories Inc., Hercules, CA, United States). The expression of the 18sRNA gene was normalized to each reaction of a single sample. The ΔΔCT method was applied for the relative gene expression.

**TABLE 2:** All primers sequence for qRT-PCR analysis.

Gene name	Forward	Reverse
18sRNA	GAC​ACG​GAC​AGG​ATT​GAC​AG	GCT​CCA​CCA​ACT​AAG​AAC​GG
NFS1	GGA​GGC​CCT​ACA​AAG​TGG​AG	CCA​TCT​CCC​AGA​GAG​GAC​TCA
CARTPT	GGA​CAT​CTA​CTC​TGC​CGT​GG	CAA​TCT​GCA​ACA​CAG​CGC​C
APH1B	GGGTACCATGACAGCGCC	CCA​GCG​TCA​TGA​AAG​CTG​AAT​TA
NDUFA10	GCC​TTG​AGG​TTG​CTG​AGA​CT	GAT​GGA​ACG​CTC​CAA​GAC​CA
MRPL53	GTG​GAG​TCA​ACA​AGG​ACC​TTT​C	TGG​TTA​AGC​AGT​GAG​AAA​GAA​AAA
ATF3	CGACCAACCCGCGCTC	CTC​TCC​AGT​TTC​TCT​GAC​TCC​TTC​T
CBLN1	AGA​CCA​TCC​AGG​TGA​GCC​T	AGG​AAC​CAT​AAT​GAC​AAG​GCA
MT1	CTG​CTG​CCC​TCA​GGT​GTA​AA	ATG​CTC​GGT​AGA​AAA​CGG​GG
OXTR	CTG​CTG​TGT​CGT​CTG​GTC​AA	GCT​TGA​CAC​TAC​TGA​CCC​GT
LOC102549072	GTC​CTT​TCA​GTC​TTG​CGG​GA	CCT​AAG​TCC​TCC​GAC​CCA​GA
LOC103693876	GTA​GAA​CCC​ACA​CGC​AGG​AA	AGA​AGC​AGG​CAA​ATT​CCC​GA
LOC102550954	ACC​ATG​GTC​AAT​GGC​ACT​GT	TCT​GGA​ACC​AGG​ACT​GAC​CA
LOC108351425	TGG​GAA​CCA​GAA​CCC​AAA​TGT	TGG​CAG​TGA​TTG​GTA​TTG​AGG
LOC103691475	TGA​GGG​GGT​TAA​TCA​GGT​GT	TGC​ACT​CTT​TTG​GTG​TCA​GT
LOC108352208	GAG​GTT​CAG​GAA​GAG​CAC​CAG	AAC​AGG​AGC​TAC​CCA​CCA​AGA​G
LOC103694103	GGC​TTC​AAA​AGG​CTG​TGG​TG	GTG​GAG​GTG​TGT​CAG​CAG​TT
LOC103693799	TGA​TGA​ACA​GGG​GCA​TCT​GAA	GCA​CAC​CCT​ACA​TCT​CAA​GCA
GPX4	CAA​AGT​CCT​AGG​AAG​CGC​CC	GCA​TCG​TCC​CCA​CTT​ACA​CA
SLC7A11	GTG​TTT​GCT​GTC​TCC​AGG​TTA​T	TCT​TTA​GAG​TCT​TCT​GGT​ACA​ACT​T
SLC1A5	GGG​CTG​TAG​GAT​GAC​AGG​AAT	GTC​CCG​AAA​GCT​GTA​GCC​AG
PTGS2	ACG​TGT​TGA​CGT​CCA​GAT​CA	ACG​TGG​GGA​GGG​TAG​ATC​AT

### 2.9 Western Blot

Western blot was used to determine the protein expression levels in the hippocampus corresponding to the identified genes. The tissues were suspended in lysis buffer containing 50 mM Tris (pH 8.0), 150 mM NaCl, and protease inhibitors and further sonicated for 30 s at 10% amplitude (ultrasonic cell crusher, Ningbo) to sufficiently dissolve the total proteins. The total proteins in the supernatant were collected using a 16,000 g centrifuge at 4°C for 20 min. The protein concentration of supernatants was determined using a BCA assay kit (Beyotime, Nanjing, China). Protein samples were separated on polyacrylamide gels with a 15% polyacrylamide content and then transferred to PVDF membranes (0.45 mm, Millipore, Bedford, MA, United States). The membranes were blocked for 1 hour at room temperature with 5 percent nonfat milk (BD Biosciences) in Tris-buffered saline with 0.1 percent Tween (TBST), then incubated overnight at 4°C with the primary antibodies anti-ATF3 (abs136180, Absin), anti-GPX4 (A1933, ABclonal), and anti-SLC7A11 (PA1-16893, Thermo Fisher, United States). The membranes were treated in TBST containing 5% nonfat milk for 1 hour at room temperature with horseradish peroxidase-conjugated anti-Rabbit IgG (Goat mAb, Abcam, ab6721). The Enhanced Chemiluminescence Kit (Thermo Pierce, Waltham, MA, United States) was used to detect the immunoreactivity, which was then viewed using ImageJ (BIO-RAD, United States). The -tubulin expression levels were used to normalize the sample’s expression levels.

### 2.10 Protein-Protein Interaction Network Analysis

The DEGs’ interactive network was built using the STRING database (http://string-db.org/). The interactions score >0.4 was used as the reliability threshold value (Otasek et al., 2019). The Cytoscape program (version 3.8.2) was used to view further and analyze the PPI network (Shannon et al., 2003). Finally, the relevance of this network was determined by calculating the connectivity degree of each protein, which is the number of proteins with which it interacts.

### 2.11 Competing Endogenous RNA Analysis of DElncRNAs and DEmRNAs

The miRanda database was used to predict interactions between lncRNA and miRNAs. The OmicStudio tools (https://www.omicstudio.cn/tool) were then used to determine the likely target binding of miRNA and mRNA interactions using the TargetScan and miRanda databases.

### 2.12 Rat Reactive Oxygen Species ELISA Assessment

Following the instructions, ROS was assessed via the ROS ELISA kit (Beyotime, Nanjing, China). The rats were sedated with sodium pentobarbital (40 mg/kg, i. p.) on day 21, and the rat’s entire hippocampus was promptly collected. The rats were sedated with sodium pentobarbital (40 mg/kg, i. p.) on day 21, and the rat’s entire hippocampus was promptly collected. The entire hippocampal tissue was then crushed with a sufficient amount of normal saline to create tissue homogenate. The tissue homogenate was centrifuged at 3,000 rpm for 10 min to obtain the supernatant. ROS, malondialdehyde (MDA), superoxide dismutase (SOD), and reduced glutathione (GSH) were all measured in the supernatant. For ROS detection, all reagents were prepared before starting the assay procedure according to the manufacturer’s instructions. Standards and sample diluent were added into standard and testing sample wells, respectively. After that, 100 μl of HRP-conjugate reagent was added to each test well and incubated for 60 min at 37°C. Following that, chromogen solutions A and B were added to each well, and all of the wells were incubated at 37°C for 15 min. Finally, a stop solution was applied to each well, and the Optical Density (OD) at 450 nm was determined within 15 min using a microplate reader.

### 2.13 Lipid Peroxidation Determination

The MDA level of the hippocampus was evaluated using an MDA assay kit as a marker of lipid peroxidation (A003-1, Nanjing Jiancheng Bioengineering Institute, Nanjing, China). According to the manufacturer’s instructions, standards, anhydrous alcohol, sample diluent, reagents, and 50% glacial acetic acid were all put to corresponding centrifugal tubes. After mixing, all centrifugal tubes were incubated at 95°C for 40 min before centrifuging for 10 min at 3,000 rpm to extract the supernatant. The ODs were measured at 532 nm using a microplate reader.

### 2.14 Superoxide Dismutase Activity and GSH Content Measurement

The SOD activity was measured using the SOD test kit (A001-3; Nanjing Jiancheng Bioengineering Institute, Nanjing, China). The GSH assay kit was used to determine the amount of GSH in the body (A006-2-1, Nanjing Jiancheng Bioengineering Institute, Nanjing, China). The assay was performed according to the manufacturer’s instructions.

### 2.15 Immunofluorescence Staining

On day 21, the rats were anesthetized with sodium pentobarbital (40 mg/kg, i. p.) and perfused with 0.9 percent saline (4°C) and 4 percent paraformaldehyde in PBS (4°C) via the ascending aorta. The hippocampus was then removed, fixed for 24 h in 4% paraformaldehyde, and dehydrated in a 30% sucrose solution. A frozen microtome was used to cut transverse brain sections (40 μm). The sections were first blocked in 5% normal donkey serum in PBS with 0.1% triton for 1 hour at room temperature. Next, they were treated overnight with rabbit anti-ATF3 (abs136180, Absin), anti-NeuN (66836-1-1g, Proteintech), and anti-GFAP primary antibodies (BSM-33065M, Bioss). After washing with PBS, the sections were incubated for 1 hour at room temperature with the second Cy3-, Cy5-, or fluorescein isothiocyanate (FITC)-conjugated secondary antibodies. The sections were then incubated in DAPI for 10 min at room temperature. Finally, an automatically inverted fluorescent microscope (Olympus, Japan) was used to view the sections blindly. The percentage of the stained area of each selected image was calculated using ImageJ software. Three to five images were randomly selected per rat tissue.

### 2.16 Nissl Staining

Transverse brain sections (20 μm) were prepared with a frozen microtome. The staining was performed following the manufacturer’s directions using Nissl Staining Solution (C0117, Beyotime, Nanjing, China). The sections were fixed with 4% paraformaldehyde for more than 10 min before staining and then rinsed twice with distilled water. Next, the sections were stained for 10 min with Nissl staining and then rinsed twice with distilled water. All of the sections were dehydrated with 95 percent ethanol and then treated with xylene for transparency. Finally, an automatically inverted fluorescent microscope (Olympus, Japan) was used to view the sections blindly.

### 2.17 Diaminobenzidine-Enhanced Perls’ Stain

DAB-enhanced Perls’ stain was applied as specific ferric staining. Transverse brain sections (10 μm) were prepared with a frozen microtome. The staining procedure was followed exactly as described previously (Moroishi et al., 2011). Briefly, the tissue slices were rinsed with distilled water and treated for 30 min with Perls reagent (5% potassium ferrocyanide, 5% HCl), then washed again in distilled water before incubation for 15 min with DAB (0.05% DAB in distilled water). An automatically inverted fluorescent microscope (Olympus, Japan) was then used to observe the sections blindly.

### 2.18 Iron Content Measurement

The iron content of the hippocampus and serum were measured to see if there was any evidence of iron overproduction. The measurements were performed using the tissue iron assay kit (A039-2-1, Nanjing Jiancheng Bioengineering Institute, Nanjing, China) and the serum iron assay kit (A039-1-1, Nanjing Jiancheng Bioengineering Institute, Nanjing, China). The tests were carried out under the manufacturer’s instructions. The ODs were measured at 532 nm using a microplate reader.

### 2.19 Statistical Analysis

GraphPad prism 9.0 was used for statistical analysis. For comparisons between two groups, the Student’s t test was used. When comparing the population mean of various related groups, ANOVA tests are acceptable. In graphs, all data is provided as means ± SD. If *p* < 0.05, the comparison is regarded as statistically significant.

## 3 Results

### 3.1 Establishment of the Rat Model of Chronic Constriction Injury Induced Memory Impairment

**FIGURE 1:**
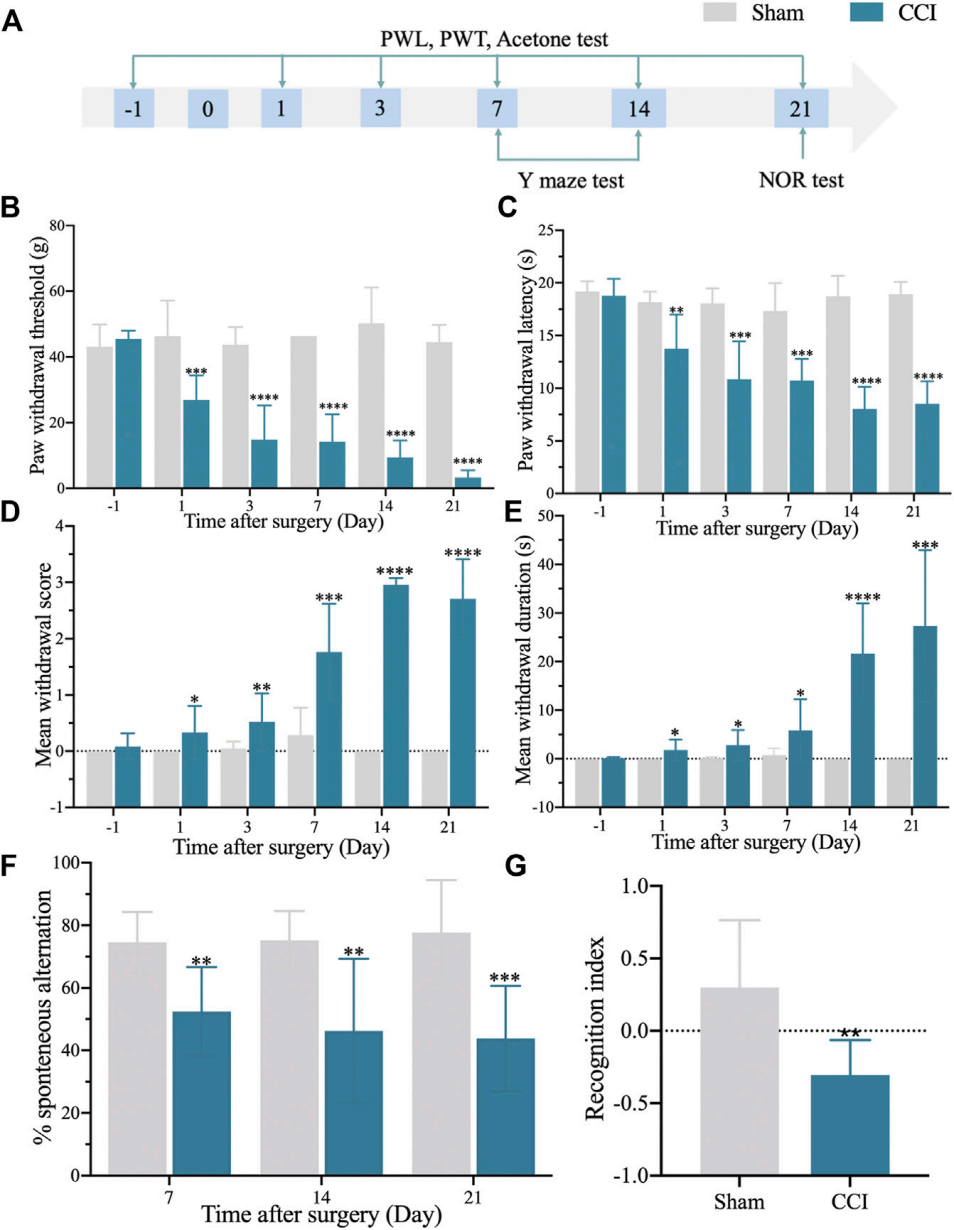
The rat model of CCI exhibits persistent mechanical and thermal hypersensitivities and accompanied by long-term memory deficit. **(A)**: The flowchart of the establishment of the CCI-induced memory impairment rat model. **(B)**: 50% paw withdrawal thresholds (PWTs) of the left hind paw of sham and CCI-induced memory impairment rats. **(C)**: 50% paw withdrawal latencies (PWLs) of the left hind paw of sham and CCI-induced memory impairment rats. **(D,E)**: Mean withdrawal scores and duration of cold hyperalgesia. **(F)**: %spontaneous alternation in the Y maze test. **(G)**: Recognition index in the NOR test. n = 6–9 rats/group. *p < 0.05, **p < 0.01, ***p < 0.001, ****p < 0.0001 vs sham group. Student’s t and ANOVA tests were used for comparisons.

We started by establishing a CCI rat model with memory impairment. After that, the flowchart was used to look at nociceptive thresholds and cognitive activities after CCI (Figure 1A). PWT and PWL decreased dramatically and consistently in the CCI group (Figures 1B,C). In addition, the CCI group developed prolonged cold hyperalgesia (Figures 1D,E). In the Y maze test, spontaneous alternation was considerably reduced on day 7 after CCI compared to the sham group and continued until the conclusion of the observation period (day 21) (Figure 1F). After CCI, the discrimination index in the NOR test was considerably lower on day 21 (Figure 1G). These findings revealed that a successful model of CCI with a memory problem had been established.

### 3.2 Transcriptome Profiling of Hippocampus of Chronic Constriction Injury Rats With Memory Impairment by RNA-Seq

We harvested the hippocampus of CCI with memory impairment rats and sham rats to investigate the causes of CCI-induced memory impairment. RNA-Seq was then used to examine the mRNA and lncRNA expression profiles. The sequencing yielded over 95 million raw reads per sample, with a clean reads Q30 ratio of almost 93.0% (Table 1). RNA-Seq resulted in the mapping and identification of 22,601 mRNAs and 15,683 lncRNAs. We then set the filtering criterion for the DEGs to be *p*-value 0.05 and |Log2 (fold change)| 0.58, as seen in the volcano figure (Supplementary Figure S1A, B). As a result, 179 DEmRNAs and 191 DElncRNAs have been discovered (Supplementary Figure S1A, B and Supplementary Table S1, B). We next used a heat map to describe the DEGs we found, followed by hierarchical clustering analysis. There was a substantial distinction between the sham and the CCI groups with memory impairment. In addition, the results showed clear segregation between the sham and CCI with memory impairment groups (Supplementary Figure S1C,D).

### 3.3 Analysis of DEmRNAs and DElncRNAs in the Hippocampus of Chronic Constriction Injury With Memory Impairment Model Rats

Some of the DEmRNAs and DElncRNAs we found have been linked to oxidative stress or cognitive disorders, such as *SLC27A2* (solute carrier family 27 member 2, fold change = 2.332164385), *LOC108348106* (alpha-ketoglutarate-dependent dioxygenase alkB homolog 6, fold change = 3.135245512), *SLC18A1* (solute carrier family 18 member A1, fold change = 0.590955033), *NDUFA10* (NADH dehydrogenase (ubiquinone) 1 alpha subcomplex 10-like 1, fold change = 0.480971719), *BMP4* (bone morphogenetic protein 4, fold change = 0.607791806), *GDF7* (growth differentiation factor 7, fold change = 0.194844902), *OXTR* (oxytocin receptor, fold change = 2.250338978), *CBLN1* (cerebellin 1 precursor, fold change = 2.247821962). There were 25 DEmRNAs with expression changes greater than 5-fold, with 13 upregulated mRNAs (such as *LOC100911730* with 54.98731663 of fold change) and 12 downregulated mRNAs (such as *LOC685716* with 0.025834514 of fold change). *TCONS_00012750* (fold change = 288.6572008), *TCONS_00012029* (fold change = 226.3665341), *TCONS_00030575* (fold change = 0.004728013), *TCONS_00026782* (fold change = 0.00562614) were among the 46 upregulated genes and 32 downregulated genes identified in the DElncRNA profile. Tables 3, 4, 5 and 6 describe the Top 20 up- and down-regulated genes in further detail.

**TABLE 3:** The detail of the top 20-upregulated DEmRNAs.

Upregulated gene name	Gene ID	Location	Log2 (fold change) (CCI/Sham)	*p* value
LOC100911730	64315	196564744–196568753	5.78102698	0.01230071
Tsen34l1	100909510	63242432–63250265	5.57491881	0.00877734
Six2	366542	8974859–8981345	4.97072558	0.02731944
LOC100912573	293749	208243561–208255400	4.5235508	0.03086266
Yipf7	364147	38571045–38599070	4.3849271	0.03951931
Adam5	498654	67223095–67298733	3.96381527	0.02477011
Fkbp6	288597	21318251–21390350	3.25554386	0.00830697
Tnnt1	171409	69306362–69316721	3.20696783	0.00022016
RGD1562652	499402	2632898–2635694	2.64444974	0.0097335
LOC103690118	83836	839788–883946	2.63854731	0.0103477
LOC100909392	292944	12698107–12775561	2.60782392	0.0000251
Ces1d	113902	13873490–13912035	2.48941746	0.04505145
Tbcb	292777	85477639–85483488	2.35883642	0.0000165
Phykpl	100169747	35839965–35863631	2.25983594	0.0000411
Prg4	289104	62487257–62504657	2.25639342	0.02979234
LOC100912228	24604	78881294–78888495	2.24267013	4.61E-08
LOC100911186	140547	194895036–194903863	2.20391882	0.00244014
Pnpla2	361676	196552723–196557805	2.1788874	0.000012
Hprt1	24465	132736175–132768149	2.08657003	0.00037877
LOC685933	685933	236603597–236685859	2.02708822	0.03049839

**TABLE 4:** The detail of the top 20-downregulated DEmRNAs.

Down-regulated gene name	Gene ID	Location	Log2 (fold change) (CCI/Sham)	*p* value
LOC685716	685716	55279853–55326753	−5.2745565	0.00383337
Slc24a5	311387	112319305–112339231	−5.140615	0.01109464
LOC100912034	64199	32023003–32028443	−4.9495918	0.0065692
P2ry10	317219	72121558–72207174	−3.8438431	0.03376527
LOC100910278	64040	79505738..79522539	-3.6968195	0.00012598
LOC102557478	102557478	68443437–68505774	−3.2566427	0.01336716
Mrpl53	362388	115615329–115616219	−2.6079781	1.58E-05
NEWGENE_1308196	301352	40125352–40136189	−2.596599	0.00038693
Lipg	291437	68514923–68536105	−2.5262274	0.03772631
LOC100911814	309312	227883247..227962119	−2.4137944	0.02360257
Gimap4	286938	77636401–77643315	−2.3785594	0.0379737
Gdf7	252833	31171495–31182447	−2.3596019	0.04450489
RT1-T24-1	361787	2761541–2774749	-2.2627501	0.0416867
Kif14	360849	47926975–47990598	-2.2606806	0.03106551
Mgat2	94273	87656360–87658849	−2.1608588	0.00867277
LOC100909709	89821	96263322–96314197	−2.1581355	0.00638393
Rpl39l	497860	5455712–5459828	−2.0308892	0.02228026
Romo1	679572	144659709–144661310	−2.0164828	0.00390799
LOC100911994	312083	31484424–31585617	−1.9809055	0.00040545
RT1-Db1	294270	4548664–4558237	−1.9316996	0.00064276

**TABLE 5:** The detail of the top 20-upregulated DElncRNAs.

Upregulated gene	Gene ID	Location	Log2 Fold change (CCI/Sham)	*p* value
ENSRNOT00000076561		—	5.78512442	0.01730266
ENSRNOT00000077876		—	2.20373049	0.02111258
ENSRNOT00000079282		—	2.09798552	0.04796722
ENSRNOT00000081463		—	6.06317183	0.04165964
ENSRNOT00000083718		—	2.66877138	0.03800823
ENSRNOT00000084042		—	3.53248432	0.00746108
ENSRNOT00000086466		—	2.90999256	0.00483471
ENSRNOT00000088572		—	1.63653171	0.00185723
ENSRNOT00000088984		—	3.06189614	0.04935497
ENSRNOT00000089993		—	0.94690101	0.01452598
ENSRNOT00000092488		—	7.59302524	0.00000197
NR_126572.1	102554851	107727772–107739795	2.04684063	0.03806143
TCONS_00000046		—	3.67510588	4.54E-10
TCONS_00000063		—	3.6221488	0.00521153
TCONS_00000066		—	5.01538596	0.0000116
TCONS_00000072		—	3.72144139	7.83E-08
TCONS_00003409		—	6.19777226	0.03438166
TCONS_00004570		—	5.26965975	0.00385247
TCONS_00007059		—	1.97880803	0.00534686
TCONS_00008373		—	6.35884856	0.0057876

**TABLE 6:** The detail of the top 20-downregulated DElncRNAs.

Down-regulated gene name	Gene ID	Location	Log2 (fold change) (CCI/Sham)	*p* value
ENSRNOT00000079308		—	−6.4901256	0.00389627
ENSRNOT00000080318		—	−3.2237543	0.04966201
ENSRNOT00000080624		—	−5.5762886	0.0226858
ENSRNOT00000081988		—	−5.4100888	0.00722821
ENSRNOT00000083218		—	−1.3429282	0.00913697
ENSRNOT00000083980		—	−4.8410086	0.02553616
ENSRNOT00000085297		—	−3.4793701	0.00869084
ENSRNOT00000085430		—	−1.5983594	0.00029936
ENSRNOT00000086000		—	−5.2736508	0.00650296
ENSRNOT00000087909		—	−0.6072032	0.00820781
ENSRNOT00000090624		—	−0.9477649	0.03438361
NR_110678.1	100910558	96976181–97014187	−1.9759649	0.02172322
TCONS_00000049	—	—	−1.6223783	0.00036462
TCONS_00000050	—	—	−0.6466528	0.02698395
TCONS_00002534	—	—	−1.3421975	4.69E-08
TCONS_00002535	—	—	−0.7819754	0.00362422
TCONS_00002540	—	—	−0.7632558	0.00609918
TCONS_00002547	—	—	−2.4841896	0.02406492
TCONS_00002551	—	—	−0.9798316	0.00531827
TCONS_00002552	—	—	−1.6084354	0.00000503

### 3.4 Validation of Expression Changes of DEmRNAs and DElncRNAs via Real-Time qPCR

**FIGURE 2:**
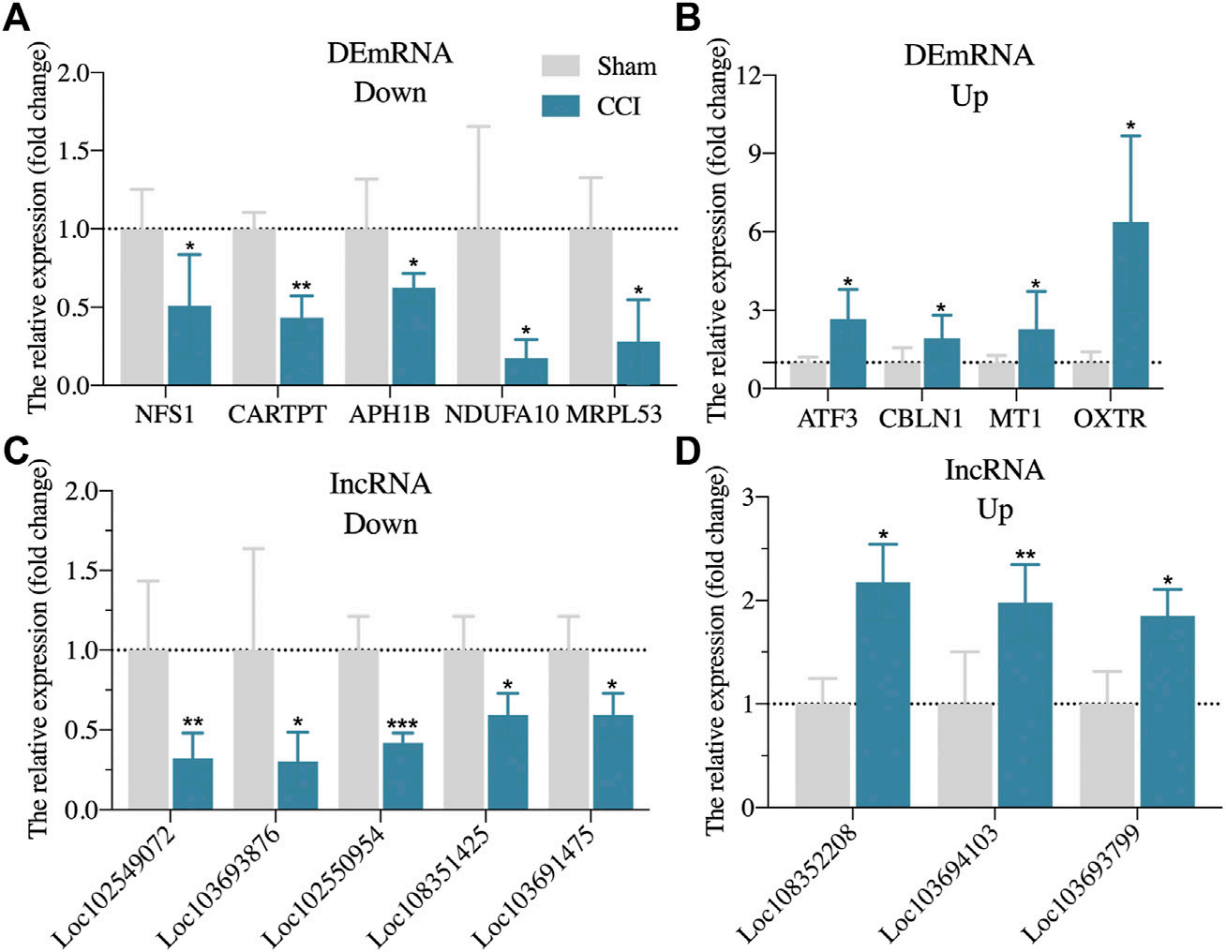
Randomly validation of DEGs from RNA-Seq via qRT-PCR. **(A)**: The expression of five randomly downregulated DEmRNAs via qRT-PCR. **(B)**: The expression of four randomly upregulated DEmRNAs via qRT-PCR. **(C)**: The expression of five randomly downregulated DElncRNAs via qRT-PCR. **(D)**: The expression of three randomly upregulated DElncRNAs via qRT-PCR. n = 3–6 rats/group. *p < 0.05, **p < 0.01, ***p < 0.001 vs sham group. Student’s t test was used for comparisons.

We also randomly reselected 9 DEmRNAs (5 downregulated and 4 upregulated) and 8 DElncRNAs (5 downregulated and 3 upregulated) from the DEGs list for qRT-PCR validation to check the correctness and reproducibility of the RNA-Seq results (Figure 2). The expression levels of *NFS1, CARTPT, APH1B, NDUFA10,* and *MRPL53* in the hippocampus of CCI with memory impairment model rats 21 days were all significantly downregulated compared to the sham groups (Figure 2A), while *ATF3, CBLN1, MT1*, and *OXTR* were all considerably upregulated (Figure 2B), which is consistent with the RNA-Seq expression profiles. Furthermore, *LOC102549072, LOC103693876, LOC102550954, LOC108351425,* and *LOC103691475* were all strongly downregulated (Figure 2C), whereas *LOC108352208, LOC103694103,* and *LOC103693799* were significantly upregulated (Figure 2D), which was also consistent with the RNA-Seq expression profiles.

### 3.5 Function and Pathway Analysis of the Identified DEmRNAs and DElncRNAs

**FIGURE 3:**
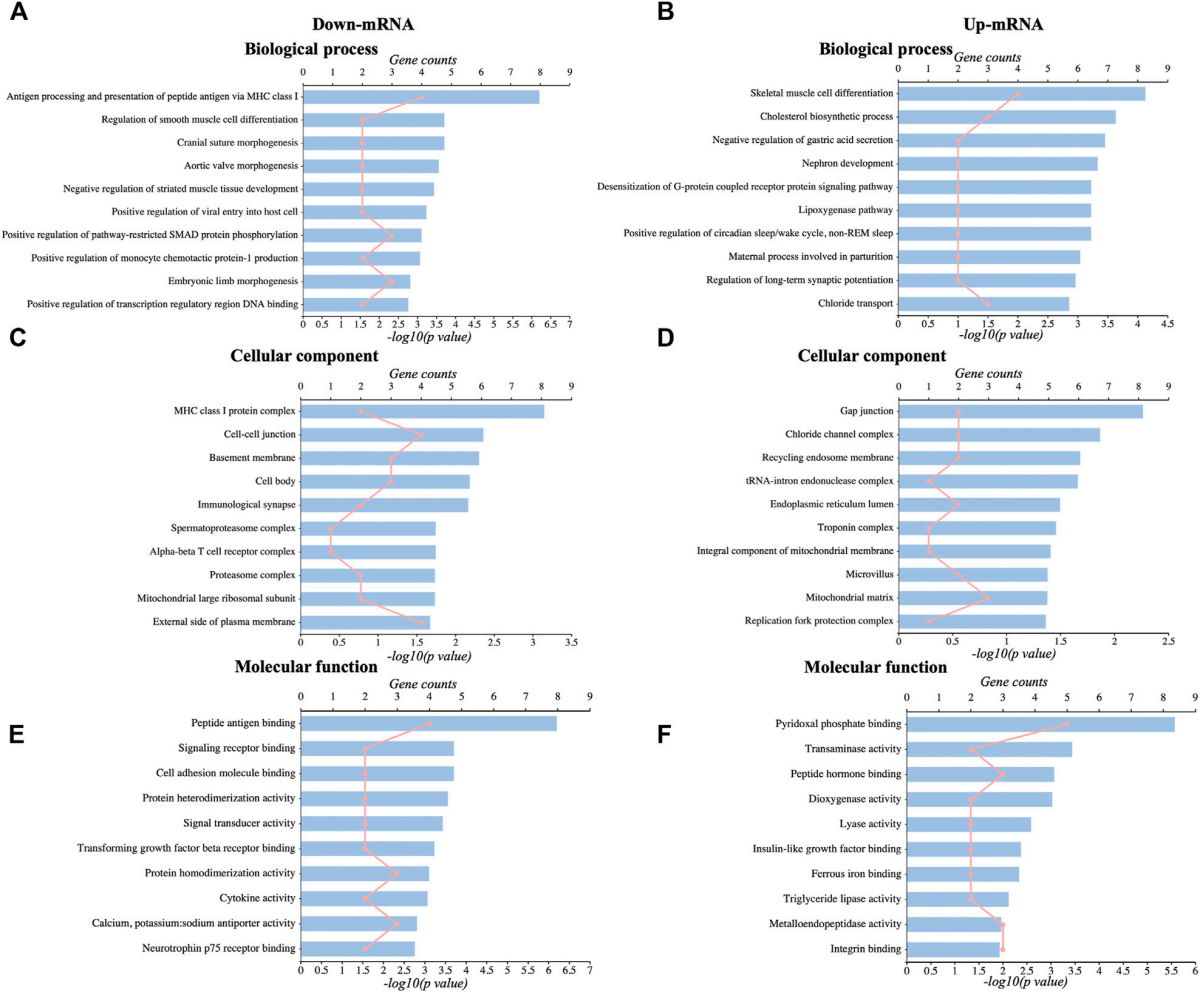
GO analysis of DEmRNAs. **(A,C,E)**: The top 10 significant biological process, cellular components, and molecular functions of downregulated DEmRNAs. **(B,D,F)**: The top 10 significant biological processes, cellular components, and molecular functions of upregulated DEmRNAs.

We used GO analysis of the discovered DEGs in the hippocampus in the sham group and CCI with the memory impairment group to discover more about the molecular process underlying CCI-induced memory impairment. The results showed that the significantly enriched BP of downregulated DEmRNAs were antigen processing and presentation, smooth muscle cell differentiation, etc. (Figure 3A, Supplementary Table S2). The significantly enriched CC of downregulated DEmRNAs was MHC class I protein complex, cell-cell junction, basement membrane, immunological synapse, mitochondrial large ribosomal subunit, etc. (Figure 3C, Supplementary Table S2). The significantly enriched MF of downregulated DEmRNAs were peptide antigen binding, signaling receptor binding, cytokine activity, etc. (Figure 3E, Supplementary Table S2). In addition, the most enriched BP of upregulated DEmRNAs were skeletal muscle cell differentiation, cholesterol biosynthetic process, regulation of long-term synaptic potentiation, etc. (Figure 3B, Supplementary Table S3). The most enriched CC of upregulated DEmRNAs was gap junction, chloride channel complex, an integral component of mitochondrial membrane, mitochondrial matrix, etc. (Figure 3D, Supplementary Table S3). The most enriched MF of upregulated DEmRNAs were pyridoxal phosphate binding, transaminase activity, lyase activity, ferrous iron-binding, etc. (Figure 3F, Supplementary Table S3).

**FIGURE 4:**
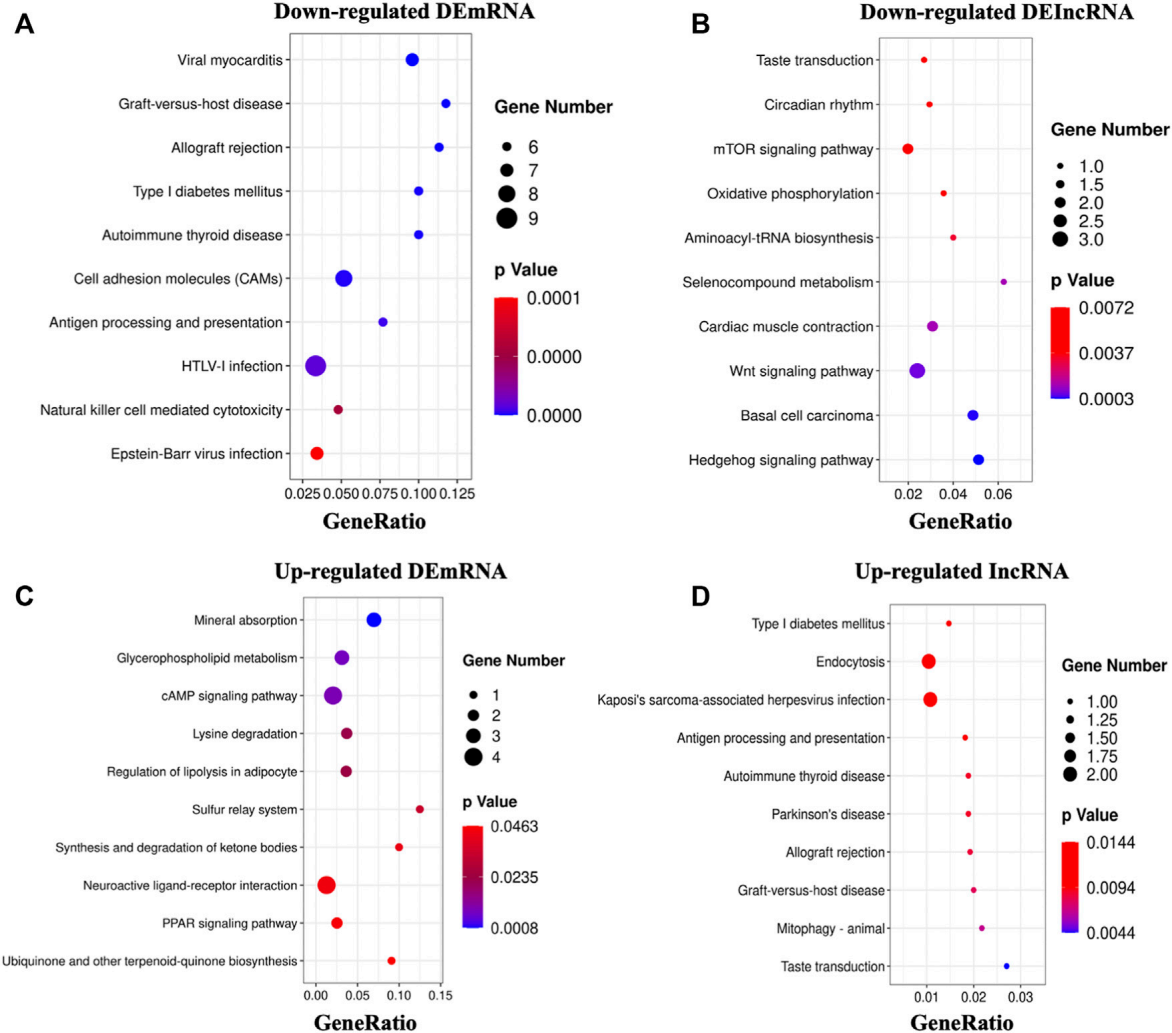
KEGG analysis of DEGs. **(A,B)**: The top 10 enriched pathways of downregulated DEmRNAs and DElncRNAs. **(C,D)**: The top 10 enriched pathways of upregulated DEmRNAs and DElncRNAs.

We then looked at the KEGG pathways of the DEmRNAs we found. The primarily enriched pathways of downregulated DEmRNAs were viral myocarditis, graft-versus-host disease, allograft rejection, etc. (Figure 4A, Supplementary Table S4). On the other hand, the primarily enriched pathways of upregulated DEmRNAs were mineral absorption, glycerophospholipid metabolism, sulfur relay system, etc. (Figure 4C, Supplementary Table S4). In addition, the potential targets of downregulated DElncRNAs mainly were enriched in the hedgehog signaling pathway, wnt signaling pathway, oxidative phosphorylation, etc. (Figure 4B, Supplementary Table S5). In contrast, the significantly enriched pathways of upregulated DElncRNAs were taste transduction, mitophagy, antigen processing, presentation, etc. (Figure 4D, Supplementary Table S5).

Then, to analyze the hub genes of identified DEGs that are involved in CCI induced memory damage, we conducted the PPI network analysis and found the significant hub genes, including *ALB, ATF3, NPY, CD3E, PIK3CA, GFAP, CD68, CFTR,* and *RT1-A2* derived from PPI analysis (Supplementary Figure S2).

### 3.6 Comparison of RNA-Seq Dataset of Chronic Constriction Injury With Memory Impairment Model Rats With Other Published Datasets of Neuropathic Pain Models and Cognitive Disorder Models

Firstly, we compared the CCI with memory impairment model rats’ RNA-Seq dataset with RNA-Seq datasets from SNI (GSE18803) and CCI models (Stephens et al., 2021). Total RNA for RNA-Seq was extracted from the spinal cord and the dorsal root ganglion. To detect DEmRNAs from SNI and CCI datasets, we used the same screening criteria as |Log2 (fold change)| ≥ 0.58*, p* < 0.05. As a result, 3 DEmRNAs from CCI with memory impairment model rats (*ATF3*, *C1QC*, and *CD68*) and 13 DEmRNAs from SNI and CCI models (*ADAMTS2*, *BCL6B*, *CLCF1*, *RBP1*, and *TPBG*, among others) overlapped with SNI and CCI models, respectively (Supplementary Figure S3A, Supplementary Table S6). Besides, no overlapping genes were found in any of the three groups.

Furthermore, we compared the CCI with memory impairment model rats’ RNA-Seq dataset with RNA-Seq datasets from aging cognitive decline (GSE9990), various stages of Alzheimer’s disease (GSE28146), and Huntington’s disease (GSE1767), respectively. We set the same screening criteria as |Log2 (fold change)| ≥ 0.58 and *p* < 0.05 to identify the DEmRNAs from the cognitive aging and AD datasets. These cognitive models did not have any genes in common (Supplementary Figure S3A, Supplementary Table S6). 11 DEmRNAs (*APOC1, TMEM106A, TSC22D4, CYR61, BCL6B, MRVI1, LAMC3, BAIAP3, CTXN3, TPBG,* and *C4B*) and 24 DEmRNAs (*ABHD10, ARC, CARTPT, CD3E, CLCF1, KIF14, OXTR, RARRES1, SLC18A1,* and *SLC2A9*, etc.) of CCI with memory impairment model rats overlapped with AD and HD model, respectively. There was no DEmRNA of CCI with memory impairment model rats overlapped with a cognitive aging model. 7 DEmRNAs (*HEYL, NFS1, MKI67, MFF, OCLN, MGAT2,* and *HPRT1*) of CCI with memory impairment model rats overlapped with both AD and HD models.

### 3.7 Construction of Competing Endogenous RNA Analysis

Based on the ceRNA hypothesis, lncRNAs may intervene in the translation of mRNAs by sponging miRNA. The ceRNA regulation network was then built in the CCI with memory impairment rat model to highlight the probable processes of lncRNA-miRNA-mRNA. By counting the number of miRNA response elements, we discovered 106 DEmRNAs, 75 miRNAs, and 90 DElncRNAs in total (Supplementary Figure S4 and Supplementary Table S7). We chose 8 DElncRNAs that were validated by our qRT-PCR to construct the network diagram involving 8 DElncRNAs, 12 miRNAs, and 58 DEmRNAs with 137 edges (Supplementary Figure S5) because the figure could not display the complex interaction (Supplementary Figure S6). The significant miRNAs competitively bound by ceRNA were rno-miR-330-5p, rno-miR-326-3p, rno-miR-326-5p, rno-miR-6334, rno-miR-540-3p, and others, according to the network analysis.

### 3.8 Chronic Constriction Injury With Memory Impairment Model Rats Showed Ferroptosis Damage in the Hippocampus

**FIGURE 5:**
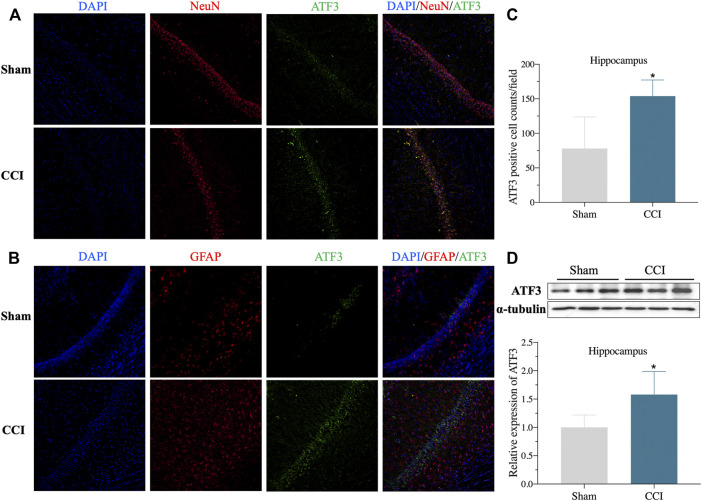
ATF3 expression of Hippocampus CA1 area increased in the CCI-induced memory impairment rat model. **(A)**: Representative immunofluorescence image indicating ATF3 antibody staining of the hippocampus CA1 **(A,B)** from sham and CCI-induced hippocampus. DAPI (blue) was used for cell nucleus labeling. NeuN (red) was used for neuron labeling. Green was used for the ATF3 positive area. GFAP (red) was used for astrocyte labeling. **(C)**: Summary of ATF3 positively stained neurons per observation area in the hippocampus from two groups. **(D)**: Validation of ATF3 protein expression in the hippocampus via western blot. n = 4–6 rats/group, *p < 0.05 vs sham group. The student’s t test was used for comparisons.

**FIGURE 6:**
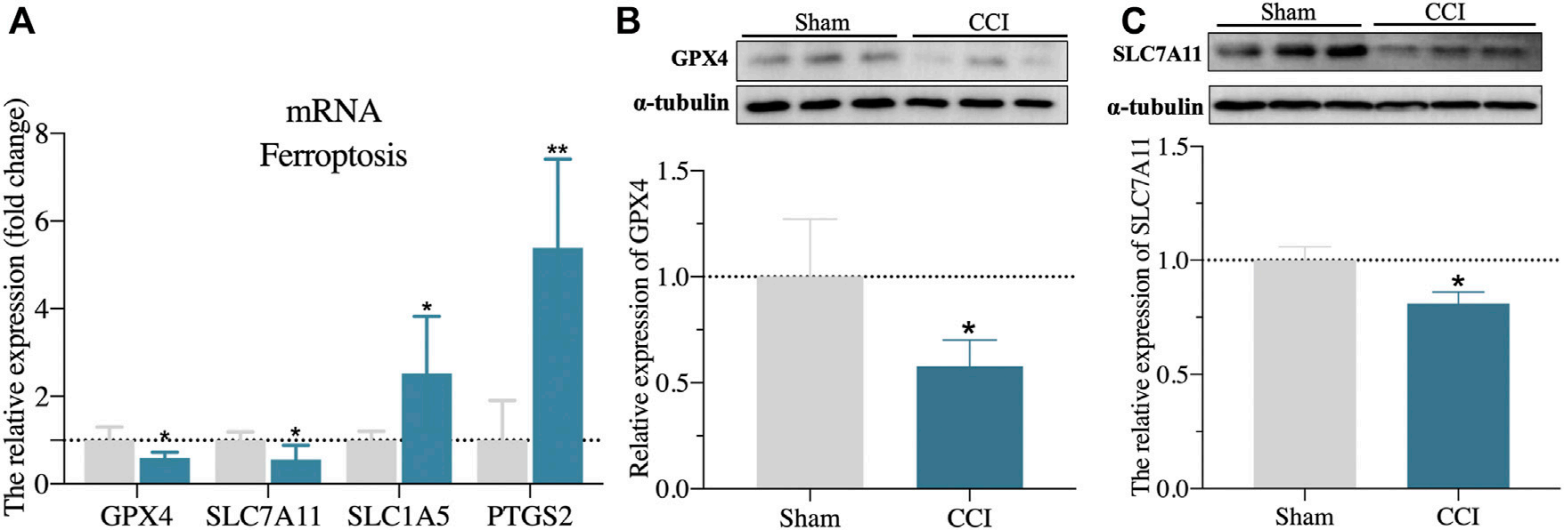
Validation of ferroptosis-associated gene markers. **(A)**: validation of gene marker mRNA levels in the hippocampus for ferroptosis by qRT-PCR. **(B,C)**: Validation of GPX4 and SLC7A11 protein expression level in the hippocampus by western blot. n = 3–6 rats/group, *p < 0.05 vs sham group. The student’s t test was used for comparisons.

The expression level of ATF3 mRNA was shown to be highly elevated in the CA1 region of the hippocampus of CCI patients with memory impairment (Figure 2B), and it has also been linked to the pathological process of pain (Ding et al., 2020). Furthermore, overexpression of ATF3 has recently been attributed to ferroptosis (Wang et al., 2020). Ferroptosis is also reported to be essential for the development of neuropathic pain (Guo et al., 2021; Wang H et al., 2021). However, it is unknown if ATF3 overproduction in the CA1 region of the hippocampus is related to memory impairment caused by CCI and the mechanism. We, therefore, performed immunostaining of hippocampus (CA1 region) using ATF3, combined with NeuN (a marker for neuron) and GFAP (a marker for astrocyte), respectively. We discovered ATF3 positively stained cells were considerably increased in the hippocampus CA1 area of CCI with memory impairment model rats 21 days compared to the sham group, as shown in Figures 5A–C. Furthermore, the number of ATF3-positive neurons rose noticeably (Figure 5A). In contrast, astrocytes showed little *ATF3* positivity (Figure 5B). We also used western blot to confirm *ATF3* expression and discovered that the level of *ATF3* expression in the hippocampus CA1 area of CCI with memory impairment model rats 21 days is considerably higher (Figure 5D). These findings suggest that *ATF3* may be linked to memory loss caused by CCI. qRT-PCR was used to examine the expression of gene markers for ferroptosis. qRT-PCR revealed that *GPX4* and *SLC7A11* gene expression were dramatically downregulated in the hippocampus of CCI with memory impairment model rats 21 days. However, *SLC1A5* and *PTGS2* gene expression were significantly elevated (Figure 6A), indicating ferroptosis damage (Tang et al., 2021).

**FIGURE 7:**
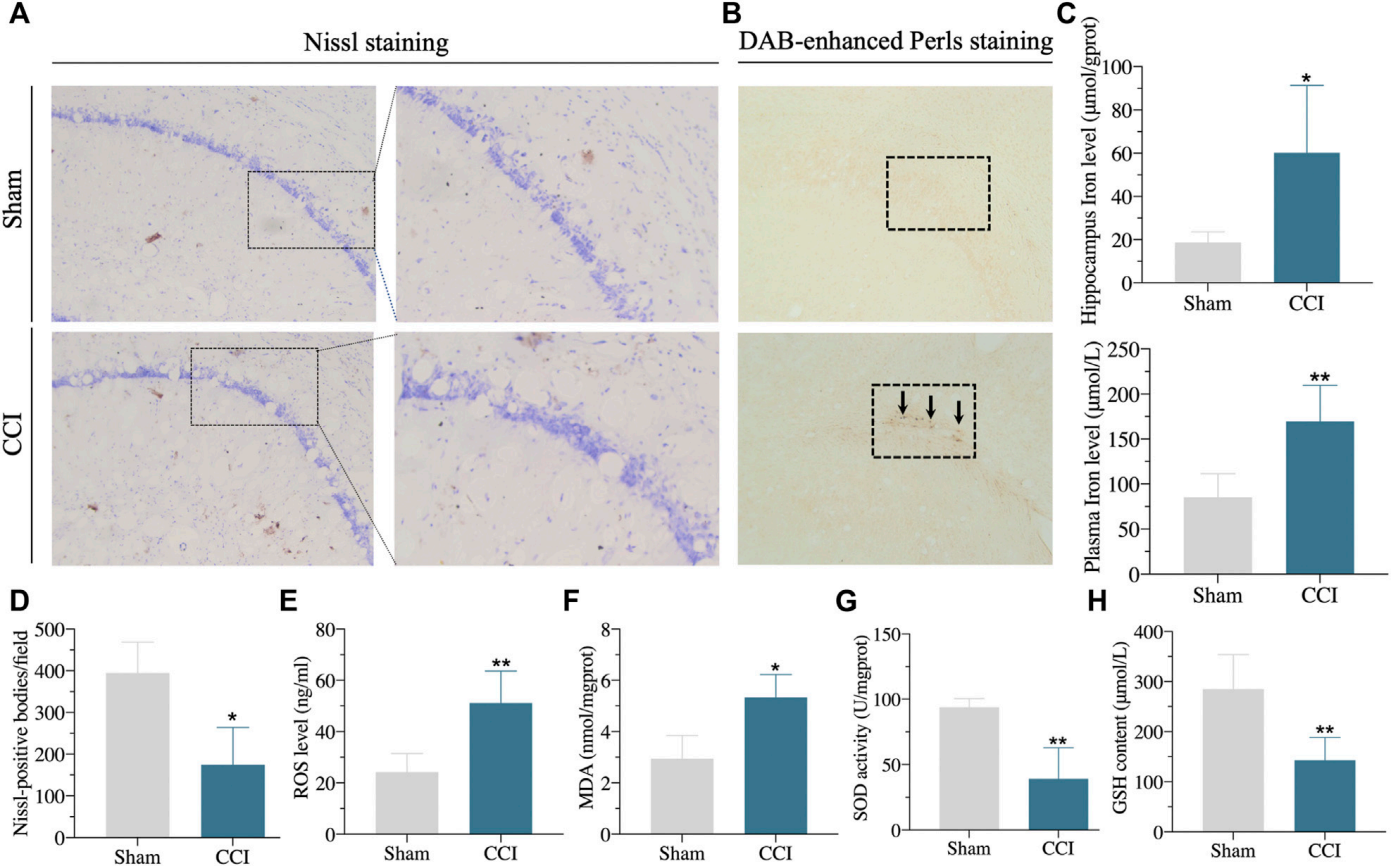
Iron overload, lipid peroxidation, and neuronal damage in the hippocampus of the CCI-induced memory impairment rat model. **(A,D)**: Compared with sham rats, the number of Nissl positive bodies in the hippocampus of CCI-induced memory impairment was decreased, and the morphology of Nissl positive bodies was obviously changed. **(B)**: DAB-enhanced Perls stain showed Perls positive iron deposits in the hippocampus of CCI-induced memory impairment rat model. **(C)**: Iron content in hippocampus and plasma were significantly increased in the hippocampus of CCI-induced memory impairment. **(E–H)**: showing the lipid peroxidation in the hippocampus of CCI-induced memory impairment as the following results: significantly increased ROS level **(E)** and MDA content **(F)**, significantly downregulated SOD activity **(G)** and GSH content **(H)** via corresponding detection kits. n = 3–6 rats/group, *p < 0.05, **p < 0.01 vs sham group. The student’s t test was used for comparisons.

Furthermore, the protein expressions of *GPX4* and *SLC7A11* in the hippocampus of CCI with memory impairment model rats 21 days were all considerably reduced, according to the western blot (Figures 6B,C). We then used DAB-enhanced Perl’s staining to detect ferric iron (Moroishi et al., 2011). Perl’s positive iron deposits were found in the hippocampus of CCI with memory impairment model mice after 21 days, implying that ferrous iron collected in the hippocampus (Figure 7B). Interestingly, the iron level of the plasma and the hippocampus were both elevated in the CCI with memory impairment group (Figure 7C).

The hallmarks of ferroptosis, including ROS, MDA, GSH, and SOD in the hippocampus, were found to include lipid peroxidation and ROS buildup. As shown in Figures 7E–H, the level of ROS and SOD were dramatically raised, whereas the GSH content and SOD activity were clearly downregulated in the hippocampus of CCI rats compared to sham rats. In addition, the number of Nissl-body neurons was significantly decreased. The shape of Nissl positive bodies was modified in the hippocampus of CCI with memory impairment group compared to the sham group, according to the results of Nissl staining (Figures 7A,D). These findings suggested that the CCI caused hippocampal ferroptosis damage.

### 3.9 Ferrostatin-1 Improved the Memory Impairment and Ferroptosis Damage of the CCI Rat Model

**FIGURE 8:**
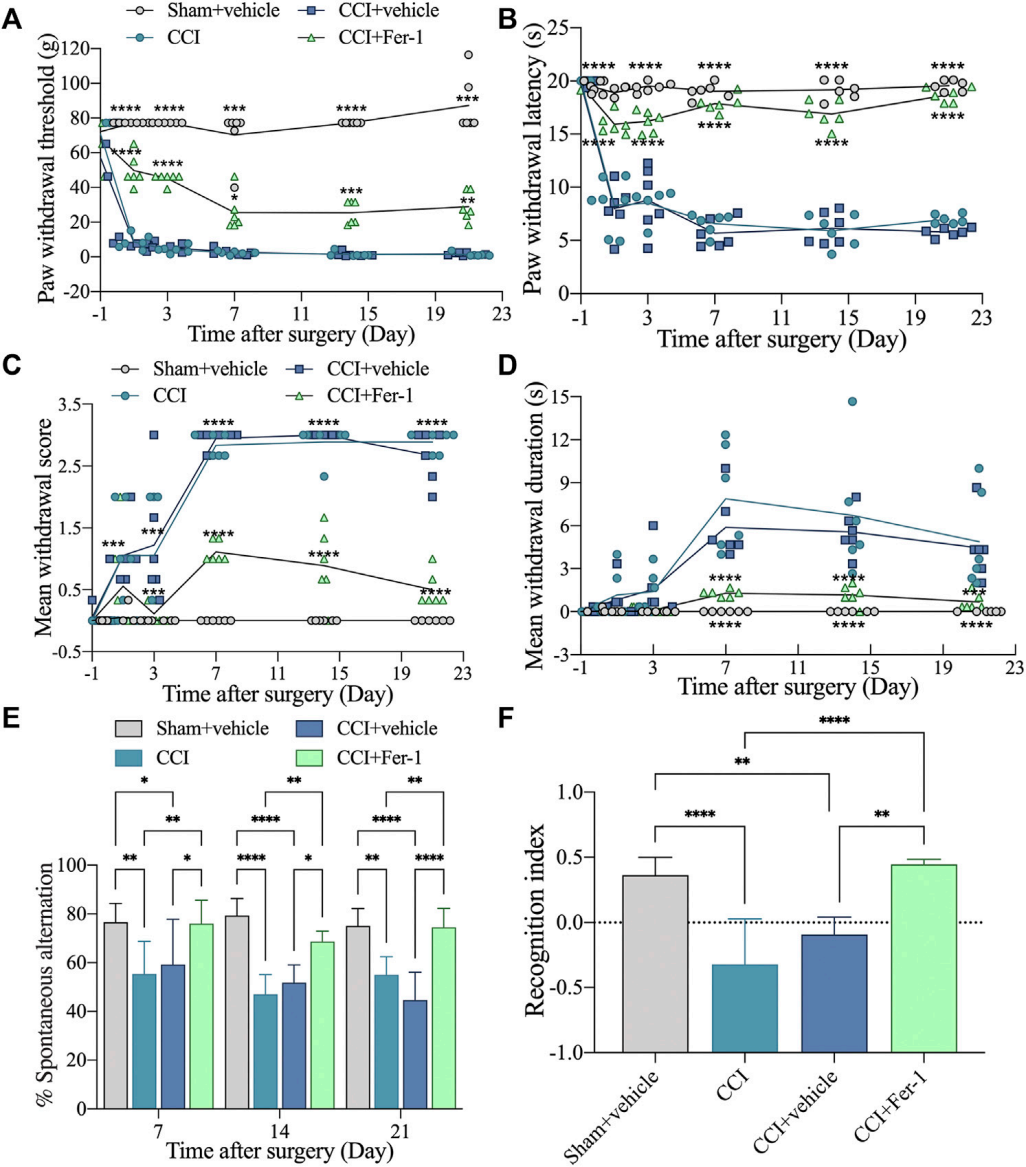
Ferrostatin-1 significantly reversed the improved CCI-induced hyperalgesia and memory impairment. **(A)**: Representative scatter diagram showing the 50% paw withdrawal thresholds (PWTs) of the left hind paw in these above groups. **(B)**: Representative scatter diagram showing the 50% paw withdrawal latencies (PWLs) of the left hind paw in these above groups. **(C,D)**: Representative scatter diagram showing mean withdrawal scores and duration of cold hyperalgesia in these above groups. **(E)**: Representative scatter diagram showing %spontaneous alternation of Y maze test in these above groups. **(F)**: Representative scatter diagram showing the recognition index of NOR test (Day 21 after CCI) in these above groups. n = 6 rats/group. *p < 0.05, **p < 0.01, ***p < 0.001, ****p < 0.0001 vs sham group or CCI + Fer-1 group. One-way ANOVA test was used for comparisons.

To verify whether ferroptosis is related to cognitive impairment induced by CCI, we employed the well-known ferroptosis inhibitor Fer-1 to treat rats. As expected, when compared to CCI or CCI + vehicle groups, Fer-1 treatment could significantly reverse the hyperalgesia generated by CCI (Figures 8A–D). Moreover, Fer-1 treatment alleviated the CCI-induced memory impairment, evidenced by greater spontaneous alternation behavior in the Y maze test and improved capacity to detect novel items in the NOR Test (Figures 8E,F).

**FIGURE 9:**
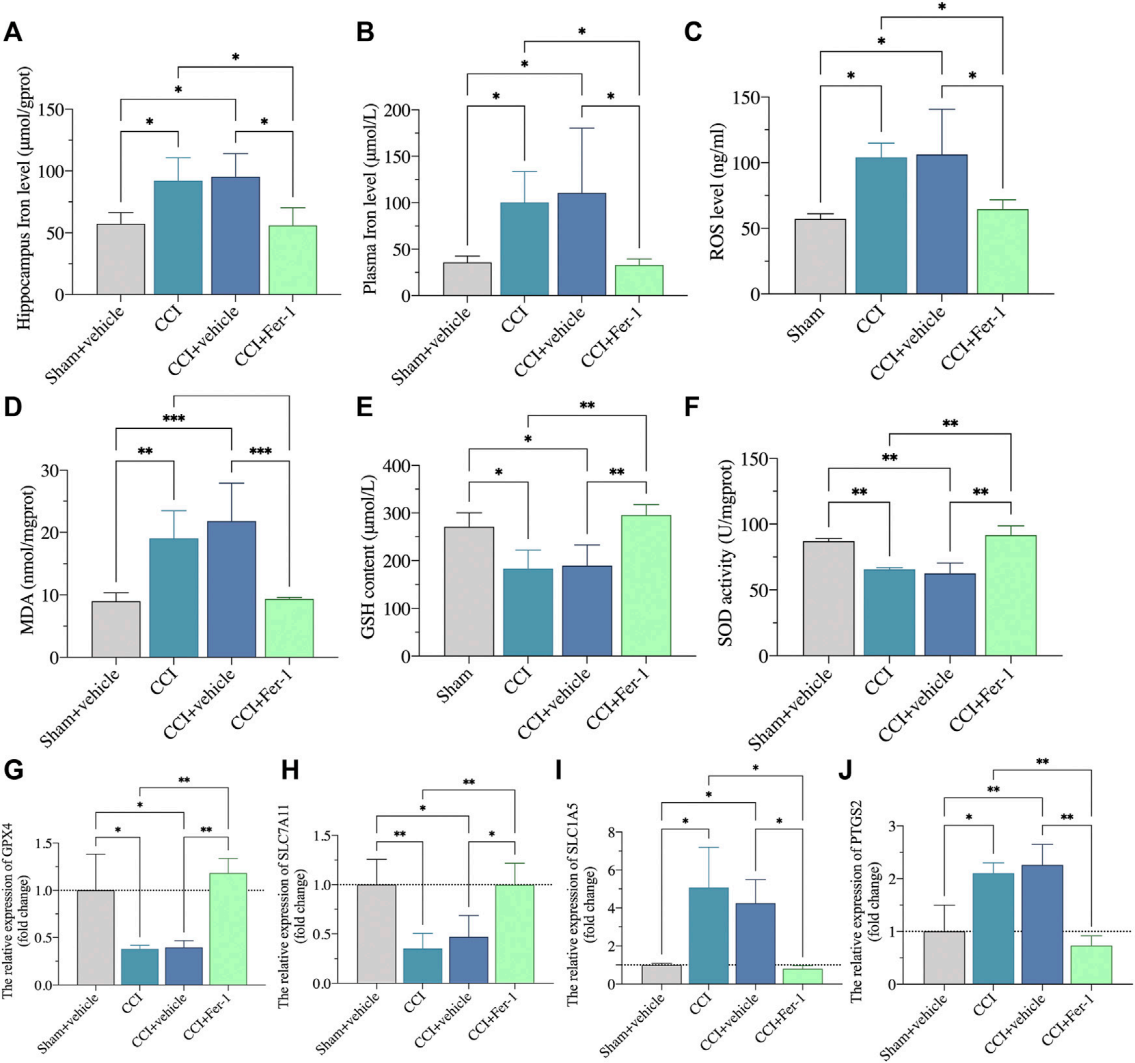
Ferrostatin-1 significantly inhibited the ferroptotic damage. **(A,B)**: Representative scatter diagram showing the iron content of hippocampus and plasma in these above groups. **(C–F)**: Representative scatter diagram showing the levels of ROS **(C)**, MDA **(D)**, GSH **(E)**, and SOD **(F)** of hippocampus in these above groups. **(G–J)**: Representative scatter diagram showing validation of gene marker mRNA levels in the hippocampus for ferroptosis. n = 3–6 rats/group. *p < 0.05, **p < 0.01, ***p < 0.001 vs sham group or CCI + Fer-1 group. One-way ANOVA test was used for comparisons.

After Fer-1 treatment, we further observed the ferroptosis damage changes of CCI rats. As shown in Figures 9A,B, Fer-1 markedly reduced the iron levels of hippocampus and plasma after CCI. Bedsides, the Fer-1 treatment reduced ROS production and lipid peroxidation while increasing GSH levels and SOD activity (Figures 9C–F). qRT-PCR revealed that in the hippocampus of CCI with memory impairment model rats 21 days after Fer-1 therapy, *GPX4* and *SLC7A11* gene expressions were dramatically increased. In contrast, *SLC1A5* and *PTGS2* gene expressions were significantly reduced (Figures 9G–J). Those results indicated that ferroptosis is involved in CCI-induced memory impairment.

## 4 Discussion

We used genome-wide RNA-Seq to assess gene expression profiles in the hippocampus of CCI-induced memory impairment model and sham rats. We identified several DEGs (mRNAs and lncRNAs) and randomly validated some of these via qRT-PCR. GO, and KEGG analysis further investigated molecular functions, cellular components, biological processes, and enriched pathways. We discovered that mitochondrial-associated processes and iron metabolism were considerably enriched in a large number of DEGs. Furthermore, ceRNA analysis was used to investigate the link between DEmRNAs and DElncRNAs. This is, to our knowledge, the first study to use RNA-Seq to look at gene expression profiles and putative pathways in the hippocampus of a CCI-induced memory impairment model.

Accumulating evidence revealed that ferroptosis was a critical factor in the development of chronic pain. Ferroptosis is a novel iron-dependent cell death marked by excessive iron buildup and lipid peroxidation. Currently, ferroptosis is linked to decreased cystine/glutamate reverse transporter activity (which reduces the synthesis of antioxidant GSH), lipid peroxide accumulation (such as excessive creation of ROS), decreased *GPX4* activity, and iron overload (Chen X et al., 2021). According to recent research, iron death plays a role in the development of chronic pain. Moreover, Nissl staining is a standard marker of neuronal injury (Shao et al., 2021). The number of Nissl positive bodies in the hippocampus of CCI-induced memory impairment rats was considerably reduced, and the form of Nissl positive bodies changed, indicating hippocampal neuron injury, as compared to sham rats in this study. Furthermore, iron accumulation in the hippocampus and plasma and lipid peroxidation (overproduction of ROS and MDA, decreased SOD activity, and decreased GSH content) were all disrupted. Similarly, lipid peroxidation and gene markers (*GPX4, SLC7A11, SLC1A5,* and *PTGS2*) dysregulation of ferroptosis were discovered in the CCI-induced memory impairment model. Fer-1 is a common ferroptosis inhibitor (Dixon et al., 2012). In the present study, treatment with Fer-1 markedly reversed the mechanical, thermal, and cold pain thresholds and CCI-induced memory impairment. Besides, Fer-1 significantly reduced the iron content and lipid peroxide level (ROS and MDA). In addition, the mRNA expression of *GPX4* and *SLC7A11* were upregulated, whereas the mRNA expression of *SLC1A5* and *PTGS2* were downregulated. These findings suggest ferroptosis may be implicated in CCI-induced memory impairment.

According to GO analysis, the overexpression of DEGs in the hippocampus of two groups was highly enriched in the control of the lipoxygenase pathway and long-term synaptic potentiation. Lipoxygenase is a multipurpose enzyme that may metabolize both endogenous and exogenous chemicals, affecting the formation of reactive oxygen species (ROS) and causing intracellular oxidative stress throughout the metabolism process (Faulkner et al., 2015). Furthermore, the cell component of GO analysis revealed that one of the most significantly enriched functions in the hippocampus was mitochondrial matrix and membrane. It suggested that oxidative metabolism disorders and synaptic dysfunction could be implicated in the fundamental pathogenic mechanism of CCI-induced memory impairment. The molecular function of GO analysis DEGs was also shown to be enriched in ferrous ion binding. Iron could react with H_2_O_2_ in the Fenton reaction to form ROS due to its extensive ligand-binding and electron-transfer characteristics (Kajarabille and Latunde-Dada, 2019). Based on these findings, iron metabolism may be involved in CCI-induced learning and memory problems.

Antigen processing and presentation, mineral absorption, and the sulfur relay system were the most enriched pathways involving DEGs, according to KEGG analysis. We also discovered that one of the DEGs, *NFS1* mRNA, was drastically reduced. For the formation of mitochondrial iron-sulfur clusters, *NFS1*, an essential enzyme in eukaryotes, gets sulfur from cysteine (ISCs). In sensitive cells and certain parts of the nervous system, biogenic errors in mitochondrial ICSs cause aberrant intracellular iron distribution, mitochondrial iron buildup, oxidative phosphorylation deficiencies, and increased oxidative stress (Kajarabille and Latunde-Dada, 2019). The pathogenic process of neurodegenerative disorders such as Parkinson’s disease, aging, and Alzheimer’s disease may be influenced by a lack of iron-sulfur cluster formation and an accumulation of iron metabolism (Zecca et al., 2004; Isaya, 2014). Inhibition of *NFS1* expression was found to cause the loss of iron-responsive protein and ICSs, up-regulation of the iron hunger response (increased transferrin receptor and decreased ferritin), and an increase in intracellular free iron, all of which led to ferroptosis in a prior study (Alvarez et al., 2017). As a result, our research suggests that ferroptosis could be a critical factor in CCI-induced memory loss. The role of *NFS1* in the hippocampus in response to pain stress should be the focus of future research.

Cluster analysis revealed that the CCI-induced learning and memory impairment model was highly consistent with the sham group’s dataset and that the two groups were significantly isolated. RNA-seq is a method for predicting preliminary genes or pathways. We also used qPCR and Western blot to validate our RNA-Seq dataset at random, and we were able to identify specific genes and targets consistent with the pattern of our RNA-Seq profiles. When we analyzed our data to other well-known published datasets, we discovered certain DEGs that may be implicated in neuropathic and cognitive common pathogenic processes (neuropathic pain models and cognitive disease models). Future RNA-Seq analyses are likely to include further RNA-Seq analyses of more samples, which will be combined with our current datasets.

Increased endoplasmic reticulum stress is linked to ferroptosis, which upregulates the production of activated transcription factors (ATFs). Recent research has discovered that *ATF3* can bind to the promoter of *SLC7A11*, suppressing its expression and system Xc–transport function (which prevents lipid peroxidation and protects cells from nonapoptotic, iron-dependent death), resulting in reduced GSH production, increased free Fe^2+^, and lipid peroxidation accumulation, and ultimately causing DNA, protein, and lipid membrane damage (Wang et al., 2020; Lu et al., 2021). Furthermore, *ATF3* is a well-known marker of sensory neuron injury, with up-regulated expression found in the DRG and spinal cord in chronic pain models (Ding et al., 2020; Wang K et al., 2021). *ATF3* was highly increased in the hippocampus DEGs of CCI-induced memory impairment rats compared to the sham group, according to our RNA-Seq profiles. Using qRT-PCR, Western blot, and immunofluorescent staining, we discovered that *ATF3* mRNA and protein levels were dramatically enhanced. Moreover, we found the expression of SLC7A11 and GPX4 in the hippocampus of CCI-induced memory impairment using western blot. These findings suggested that *ATF3* may play a role in the pathological process of CNPP with cognitive impairment by preventing system Xc–and *GPX4* from causing ferroptosis.

Chronic constriction injury of the sciatic nerve is a classic neuropathic pain model (Kalman and Keay, 2014). Neuropathic pain is regarded to be a persistent stress factor that can influence alterations in various areas of the brain, including the hippocampus (Tracey and Mantyh, 2007). Neuro-immune interactions play a role in the pathophysiology of neuropathic pain caused by peripheral nerve damage. Peripheral inflammation is likely to be transferred to the brain following nerve injury via a set of well-defined immune-to-brain communication pathways (Fiore and Austin, 2016). According to a recent study, monocyte migration-mediated neuroimmune response may be linked to cognitive impairment produced by neuropathic pain (Mai et al., 2021). However, whether the occurrence of ferroptosis in the hippocampus is related to the inflammatory microenvironment, we will focus on it. In a broader sense, there are three non-exclusive channels for immune-to-brain signaling: 1) neurological transmission, 2) humoral transmission, and 3) molecular transmission (Fiore and Austin, 2016). Future studies are needed to determine if peripheral nerve damage caused ferroptosis in the hippocampus via these pathways.

## 5 Conclusion

The current study used RNA-Seq to provide a genome-wide profile of the hippocampus of a rat model of CCI-induced memory impairment. In addition, several DEGs and pathways in the hippocampus were also discovered by pathways and function analysis, which may alter ferroptosis and memory impairment in response to chronic pain stress. These findings could lead to a better understanding of the molecular mechanisms behind CCI-induced memory loss, leading to the development of new and effective treatments for CCI-induced memory impairment.

## Data Availability

The datasets presented in this study can be found in online repositories. The names of the repository/repositories and accession number(s) can be found below: NCBI BioProject accession number: PRJNA796160.
