# Supplementary material for: Transcriptome Profiles of IncRNA and mRNA Highlight the Role of Ferroptosis in Chronic Neuropathic Pain With Memory Impairment
Source: Front Cell Dev Biol. 2022 Apr 25;10:843297. doi: 10.3389/fcell.2022.843297 (PMC9082550; doi:10.3389/fcell.2022.843297)
Supplement: Supplementary file 6 [file DataSheet1.docx]

Supplementary Material

# Supplementary Tables

Supplementary Table 1. DEGs dataset.

Supplementary Table 2. GO result of the downregulated DEGs in CCI-induced memory impairment.

Supplementary Table 3. GO result of the upregulated DEGs in CCI-induced memory impairment.

Supplementary Table 4. KEGG analysis of DEmRNAs.

Supplementary Table 5. KEGG analysis of DElncRNAs.

Supplementary Table 6. Overlapping with CCI and SNI.

Supplementary Table7. ceRNA analysis.

# Supplementary Figures


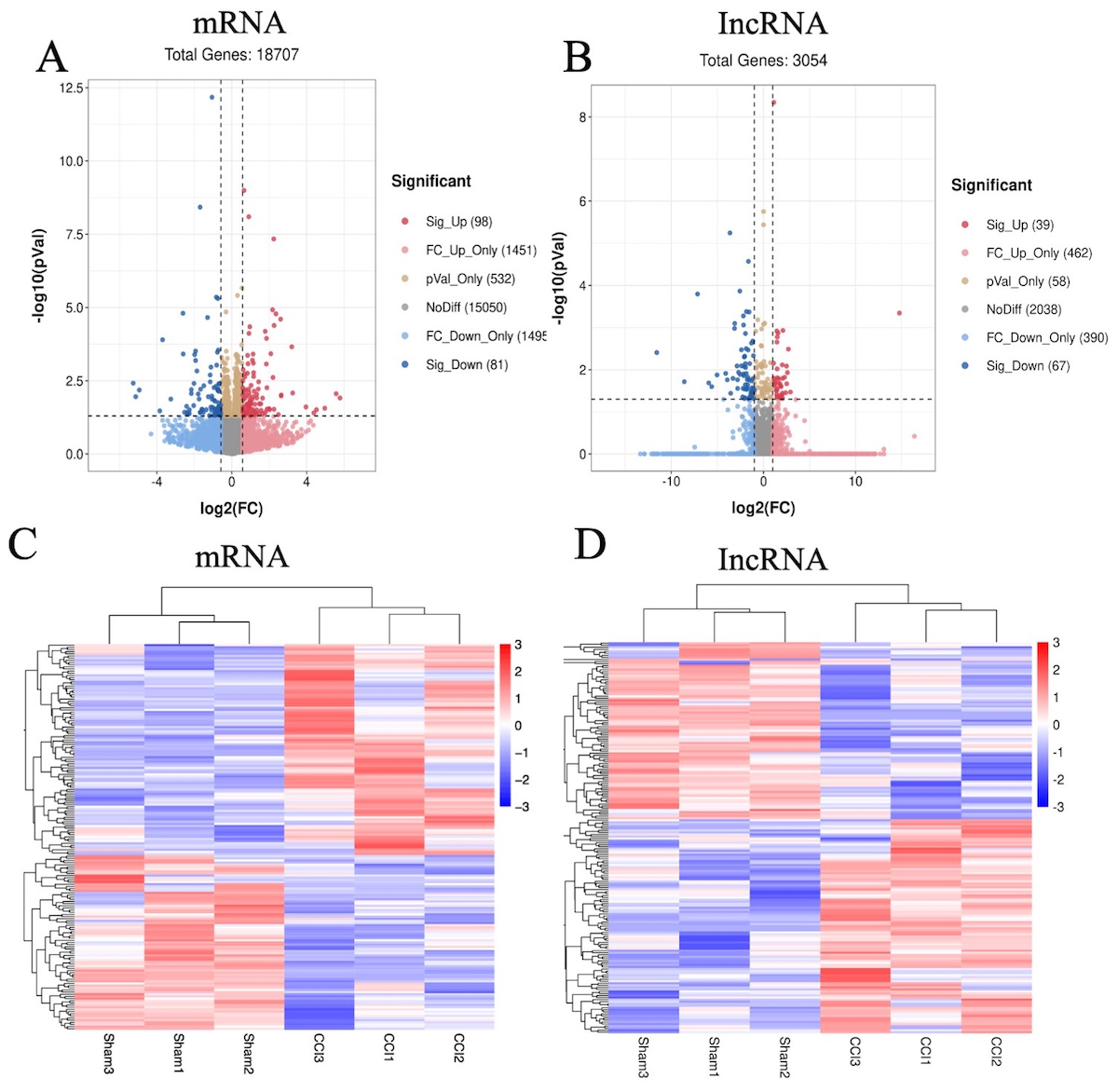


**Supplementary Figure 1**. RNA-Seq identifies expression changes of mRNAs and lncRNAs in the hippocampus of CCI-induced memory impairment rat model. A, B: Volcano plots of mRNA and lncRNA. Red and blue spots show up- and down-regulated DEGs, respectively, whereas gray spots indicate non-DEGs. C, D: Heat map showing the hierarchical clustering of DEmRNAs and DElncRNAs from sham CCI-induced memory impairment group.

**
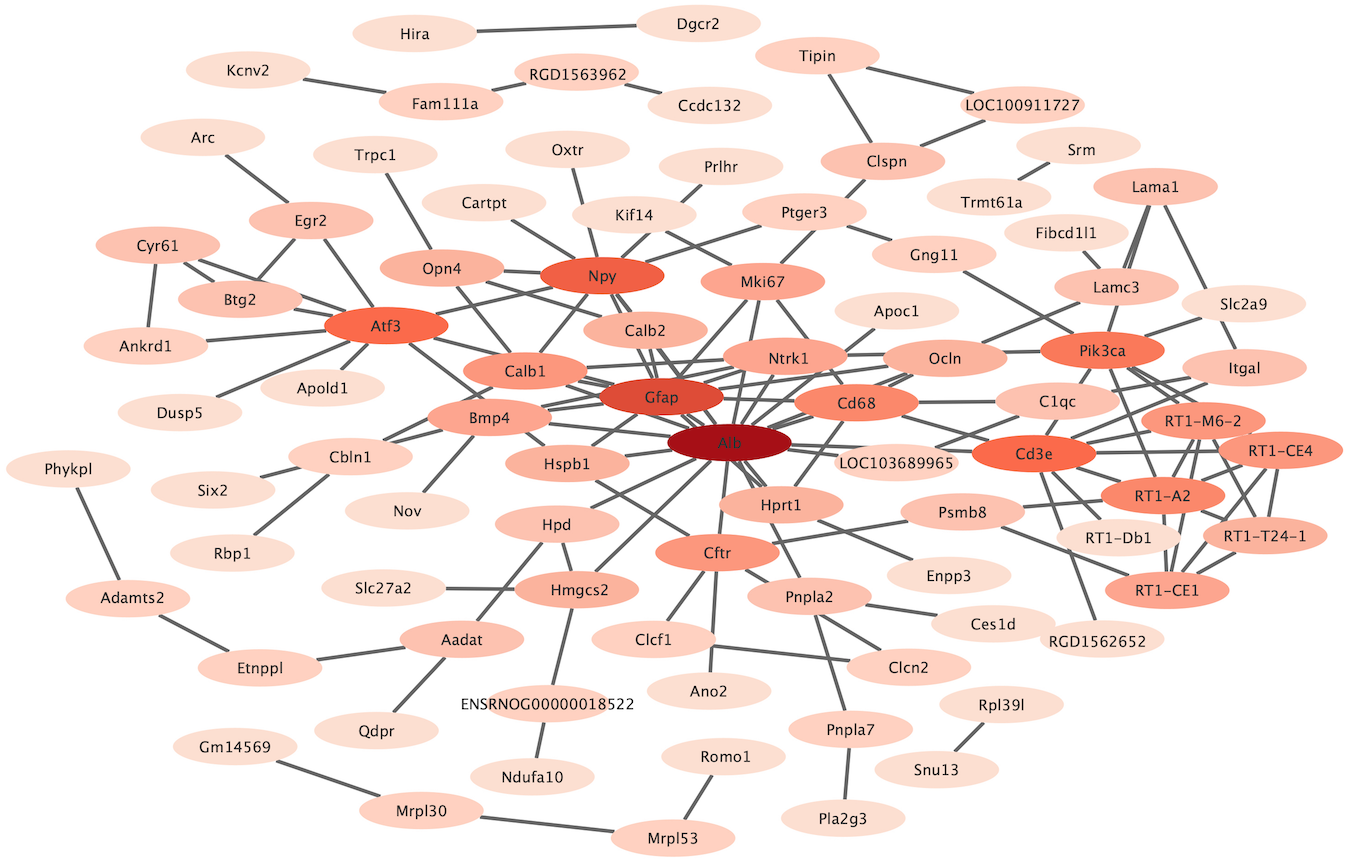
**

**Supplementary Figure 2.** PPI network analysis of DEGs in hippocampus of CCI-induced memory impairment rat model. Deep colors indicate a higher degree of interactions.


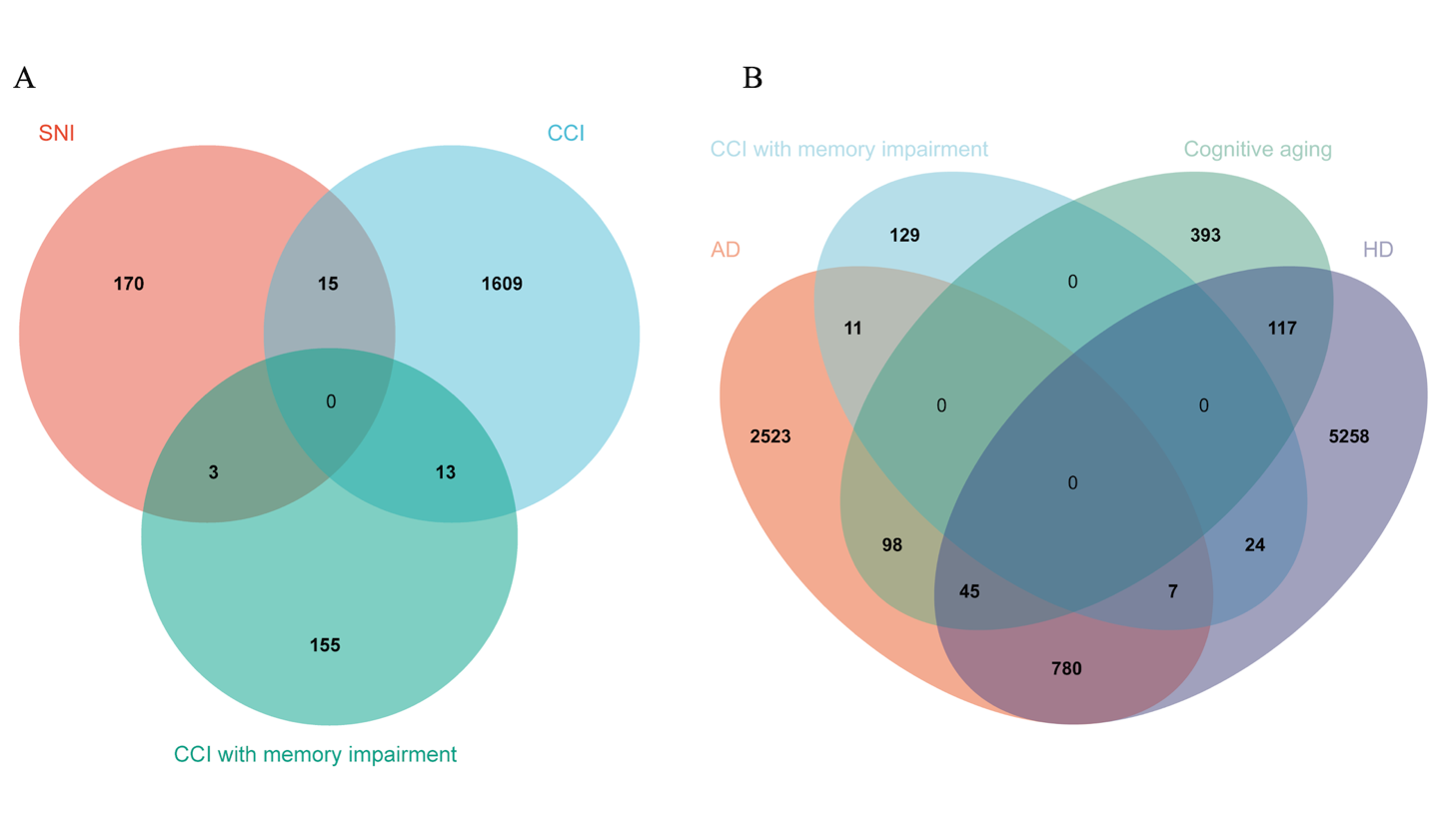


**Supplementary Figure 3.** Comparison of CCI with memory impairment RNA-Seq profiles with other published datasets of other neuropathic pain and cognitive disorder models. A: Venn diagram showing the overlapping of DEGs in the hippocampus of CCI-induced memory impairment rat model with rat model of SNI and CCI neuropathic pain. B: Venn diagram showing the overlapping of DEGs in the hippocampus of CCI-induced memory impairment rat model with other cognitive disorder models.

**
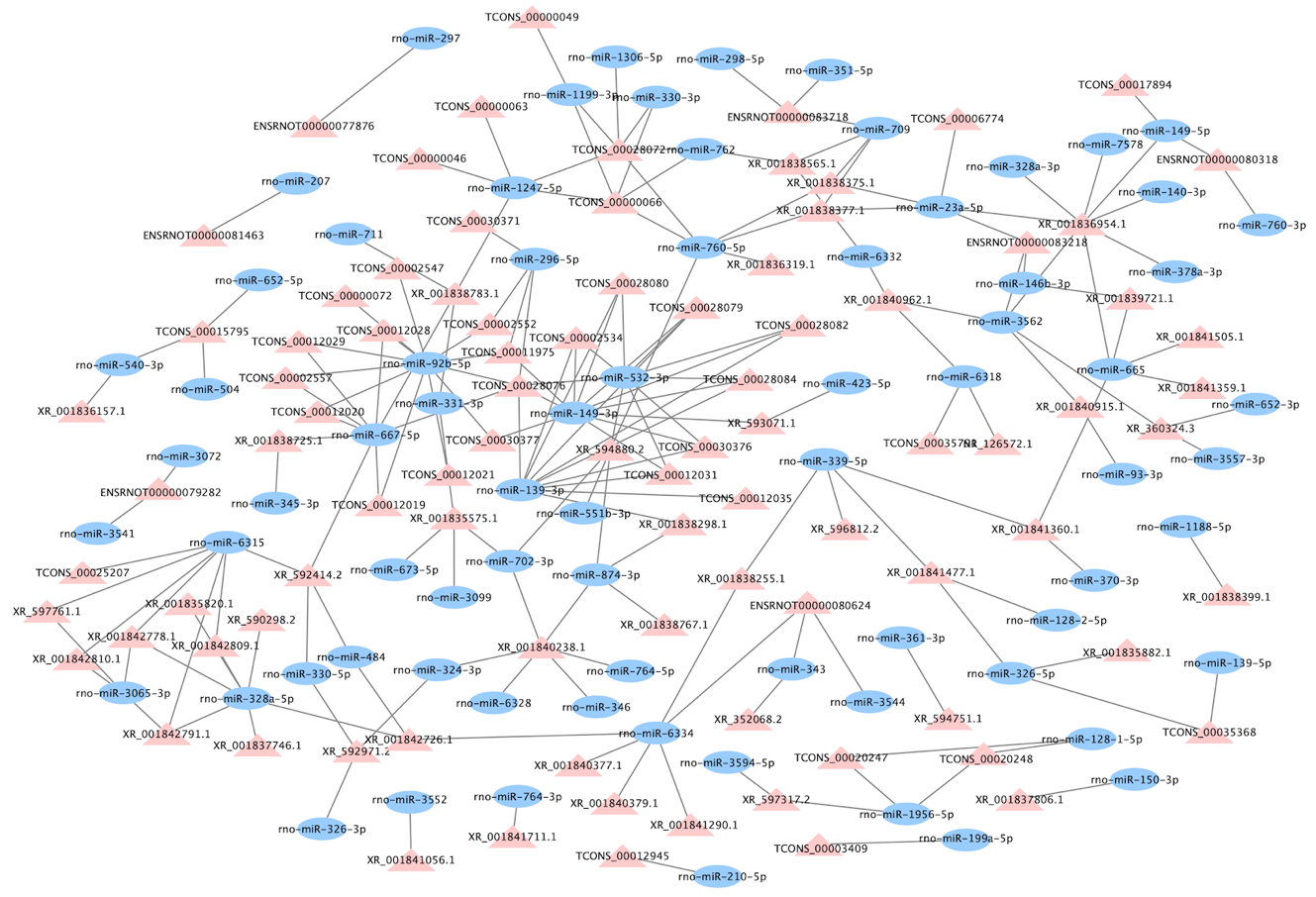
**

**Supplementary Figure 4.** The association of DElncRNAs and targeted miRNAs.


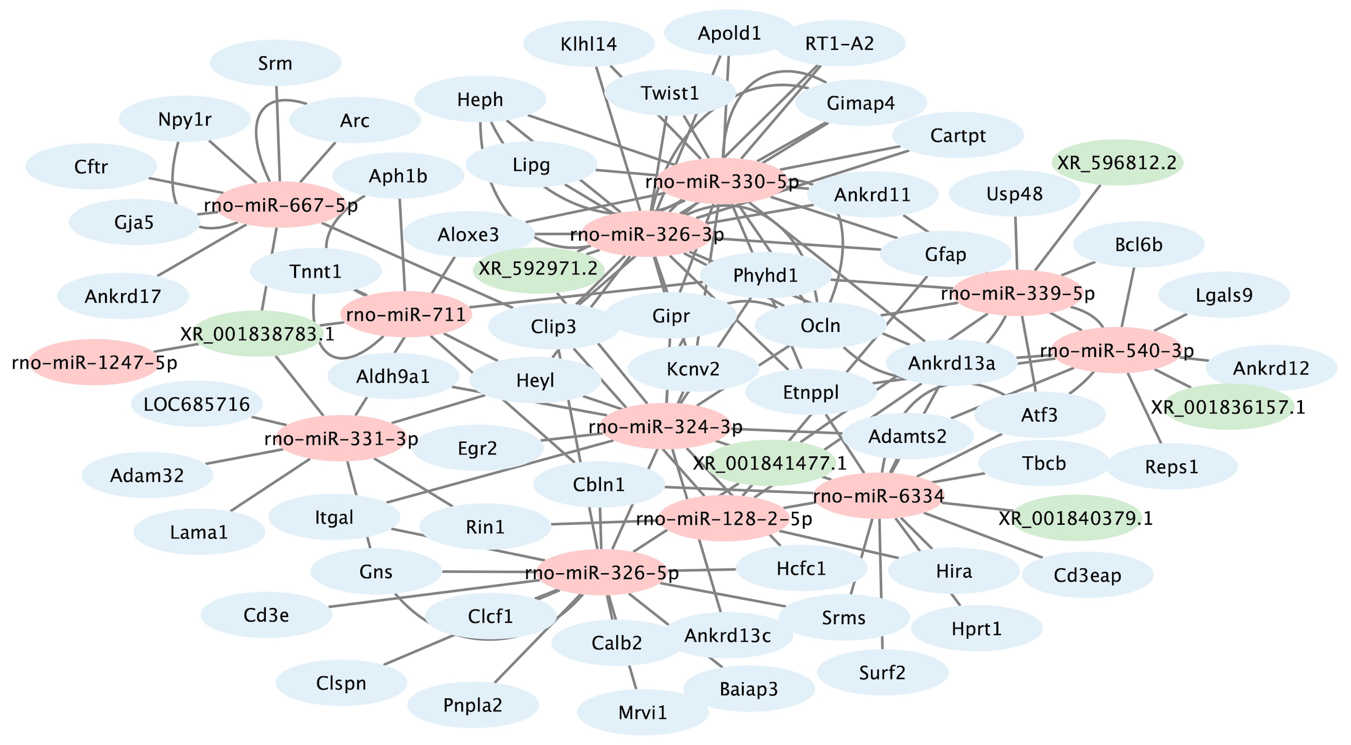


**Supplementary Figure 5.** CeRNA network analysis of the hippocampus in CCI-induced memory impairment rat model. DElncRNAs validated via qRT-PCR are selected for this analysis.


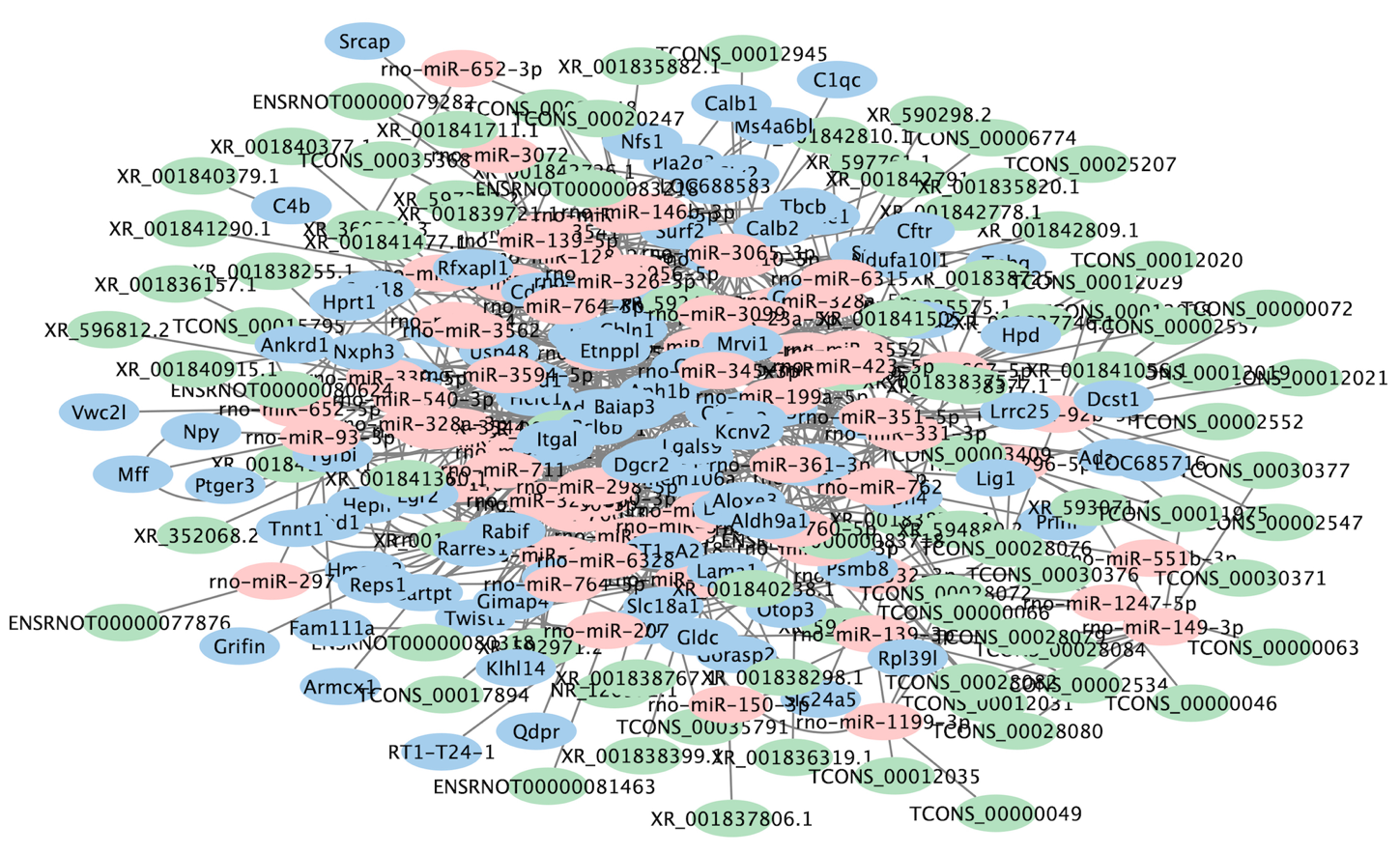


**Supplementary Figure 6.** Total ceRNA network.
